# *Streptomyces antiinflammaticus* from *Rehmannia glutinosa* Produces Dual Immunomodulatory Metabolites That Upregulate IL-10 and Inhibit GSK3β/NF-κB

**DOI:** 10.3390/molecules31213869

**Published:** 2026-10-27

**Authors:** Tong Wang, Yu Liang, Feiyan Yang, Yueqing Du, Jiaqi Li, Pengqiang Du, Yanqin Qin, Hairong He

**Affiliations:** 1 School of Pharmacy, Henan University of Chinese Medicine, Zhengzhou 450046, China; 18236169738@163.com (T.W.); liang_mail2026@163.com (Y.L.); 19547033635@163.com (F.Y.); henanlijiaqi@163.com (J.L.); 2 College of Plant Protection, Henan Agricultural University, Zhengzhou 450046, China; dyq15236015195@163.com (Y.D.); dupengqiang@henau.edu.cn (P.D.)

**Keywords:** *Streptomyces antiinflammaticus*, strain identification, compound isolation, anti-inflammatory, GSK3β/NF-κB

## Abstract

Strain RerS4 was isolated from the rhizosphere of the medicinal plant *Rehmannia glutinosa* and comprehensively identified for its taxonomic status and anti-inflammatory secondary metabolites. Polyphasic taxonomy combined with genome-based analyses, including average nucleotide identity (82.87–85.87%) and digital DNA-DNA hybridization (26.7–31.9%), confirmed that RerS4 represents a novel *Streptomyces* species, designated *Streptomyces antiinflammaticus*. Eight fermentation media were screened using an IL-6/TNF-α inhibitory bioassay in A549 and THP-1 cells, and GYM I medium with optimal anti-inflammatory activity was adopted for large-scale fermentation. Five secondary metabolites were purified from the fermentation broth, including one undescribed amide (**1**), alongside four known compounds (**2**–**5**). Bioactivity assays indicated that compounds **1** and **5** inhibited pro-inflammatory cytokine production. Mechanistically, both compounds suppressed the phosphorylation of p65 (Ser536) and GSK3β (Tyr216) in A549 cells. In THP-1 macrophages, compound **1** selectively targeted p65, while compound **5** inhibited both proteins. qPCR results revealed that compound **1** downregulated pro-inflammatory cytokines (IL-1β, IL-6, TNF-α) and upregulated the anti-inflammatory cytokine IL-10, whereas compound **5** reduced IFN-γ mRNA expression in A549 cells. This study demonstrates that *Streptomyces antiinflammaticus* is a valuable resource for distinctive anti-inflammatory and immunomodulatory natural products.

## 1. Introduction

Inflammation serves as a core pathological basis for multiple human disorders, including rheumatoid arthritis, atherosclerosis, aging-related lesions, metabolic disorders, cancer, and neurodegenerative diseases [1]. Current clinical anti-inflammatory strategies, which mainly rely on non-steroidal anti-inflammatory drugs (NSAIDs) and glucocorticoids, are frequently compromised by adverse side effects such as cardiotoxicity, hepatotoxicity, and excessive immunosuppression [2,3]. There is therefore a continuing need for novel lead chemotypes that engage well-defined intracellular targets and offer a broader therapeutic window. In this context, microbial natural products remain a privileged source of structurally and biosynthetically diverse secondary metabolites; their value as lead matter lies in this chemical repertoire, with the selectivity and tolerability of each purified isolate to be established on an individual, compound-by-compound basis rather than presumed from natural provenance alone.

Among microbial resources, actinomycetes, particularly rhizospheric *Streptomyces* associated with medicinal plants, are prolific producers of bioactive secondary metabolites with documented anti-inflammatory effects [4,5,6,7]. Multiple studies have validated their anti-inflammatory potential. For instance, actinofuranones D–I derived from lichen-associated *Streptomyces gramineus* exert prominent anti-inflammatory activity [8]; anhydroexfoliamycin from *Streptomyces* effectively ameliorates microglia-mediated neuroinflammation [9]; and xiamenmycin A from mangrove-derived *Streptomyces xiamenensis* concurrently suppresses inflammatory responses and mechanical stress, exhibiting therapeutic potential for hypertrophic scars and fibrotic diseases [10]. Importantly, a small but growing number of microbial metabolites have been shown not only to suppress pro-inflammatory cytokines but also to upregulate the anti-inflammatory cytokine IL-10, thereby exerting bidirectional immunomodulation [11,12,13]. Such dual regulation is increasingly viewed as therapeutically advantageous over conventional single target agents; however, the molecular signaling axes that underlie this dual effect, particularly those linking rhizosphere-derived *Streptomyces* metabolites to intracellular kinases such as GSK3β and downstream NF-κB signaling, remain poorly defined.

The rhizosphere of traditional medicinal plants is a particularly promising but underexplored niche. Its microbial communities have co-evolved with the host’s phytochemical defense network and are presumed to harbor biosynthetic gene clusters that have rarely been tapped [14]. *Rehmannia glutinosa* is a widely used medicinal herb with documented anti-inflammatory efficacy; nevertheless, the culturable *Streptomyces* inhabiting its rhizosphere have rarely been investigated, and neither the signaling mediators they produce nor their molecular targets have been characterized.

The present study was undertaken to close this gap by resolving the taxonomic identity, chemical output, and immunomodulatory mechanism of strain RerS4, a rhizospheric *Streptomyces* whose genome had been deposited in public databases but had never been formally classified [15]. Specifically, we aimed to (i) establish its taxonomic position through polyphasic phenotypic characterization together with genome-based ANI and dDDH analyses, formally proposing it as *Streptomyces antiinflammaticus*; (ii) screen eight fermentation media and optimized culture conditions to enable reproducible, bioactivity-guided isolation of secondary metabolites; (iii) isolate and structurally identify the active constituents, including a previously undescribed phenolic fatty amide, compound **1**, established as (*Z*)-*N*-(4-hydroxyphenyl)henicos-11-enamide, alongside a known compound **5** selected for comparative analysis; and (iv) as the central objective, interrogate their anti-inflammatory mechanisms in both epithelial (A549) and monocytic (THP-1) cell models, demonstrating that both compounds concentration-dependently suppress NF-κB activation and downstream pro-inflammatory cytokines, whereas GSK3β modulation is compound- and cell-type-dependent; notably, the new compound **1** additionally upregulates IL-10, thereby achieving bidirectional immunomodulation.

Taken together, this work formally describes a genome known but taxonomically unresolved rhizosphere streptomycete, characterizes a new phenolic fatty amide chemical probe alongside a known congener, and most significantly, provides the first experimental evidence that *R. glutinosa* rhizosphere-derived *Streptomyces* metabolites act on the NF-κB signaling axis, with the new compound **1** additionally upregulating IL-10 to achieve bidirectional immunomodulation. These findings support further exploration of medicinal plant rhizosphere *Streptomyces* as sources of anti-inflammatory leads.

## 2. Results

### 2.1. Characterization of Strain RerS4

**Figure 1:**
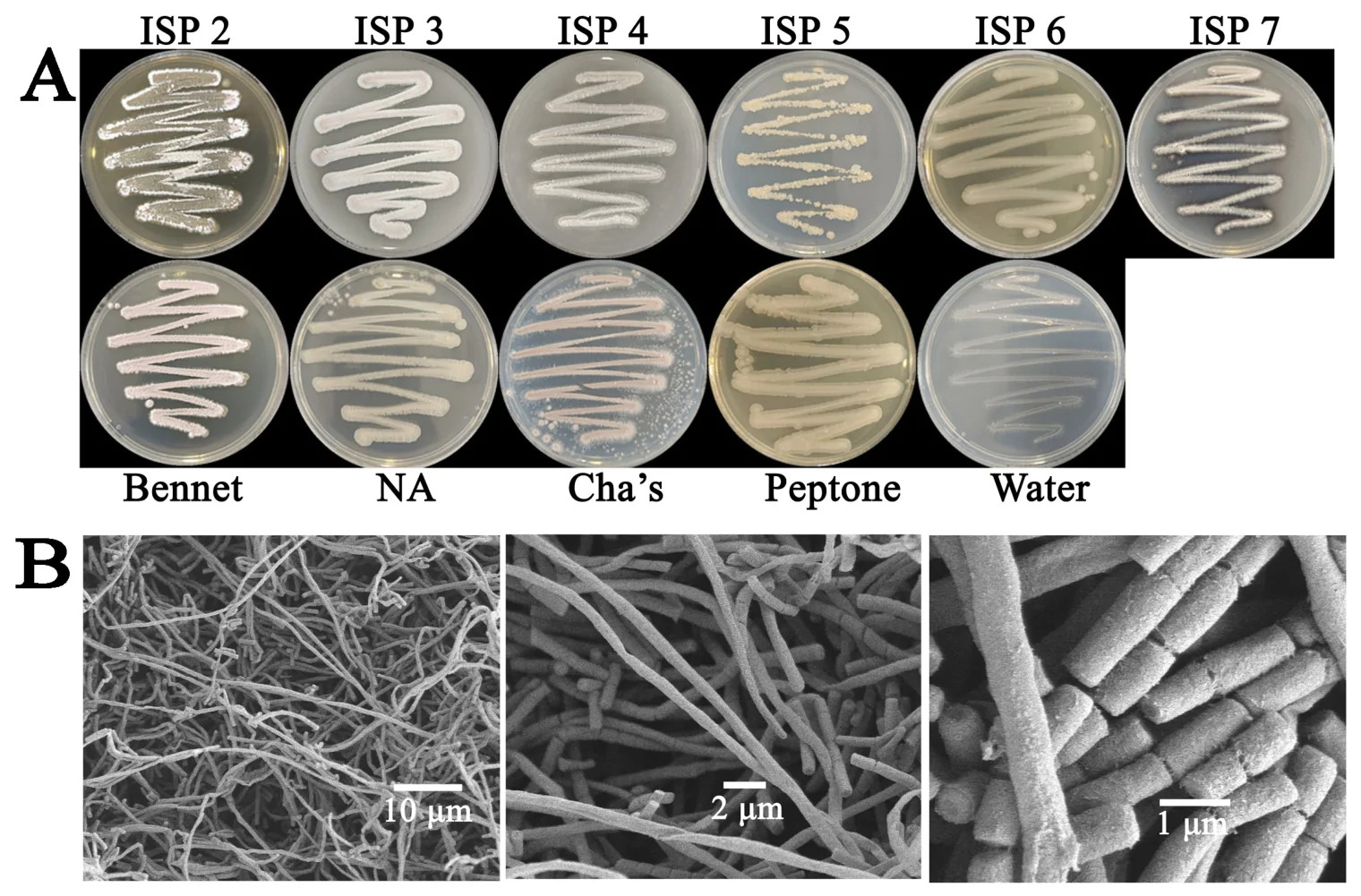
Morphological characteristics of RerS4. (**A**) Colony morphology on different media; (**B**) scanning electron micrograph showing hyphal morphology and spore chain arrangement.

Strain RerS4 is a Gram-positive, aerobic, and non-motile actinobacterium. It exhibited robust growth on ISP 2, ISP 3, Bennett’s, and Cha’s agar media, moderate growth on ISP 4, ISP 6, ISP 7, nutrient agar, and peptone media, and poor growth on ISP 5 and water agar. Colony color was determined according to the Inter-Society Colour Council-National Bureau of Standards (ISCC–NBS) color system [16]. Strain RerS4 produced a dark brown pigment on ISP 7 medium. The aerial mycelium appeared sandy on ISP 2, white on ISP 3, ISP 4 and ISP 7, and light pink on Bennett’s and Cha’s media (Figure 1A). The substrate mycelium displayed a golden-yellow color on ISP 2 and ISP 5, beige on ISP 3, ISP 4 and ISP 7, pale yellow on ISP 6, nutrient agar and peptone media, and pink on Bennett’s medium (Table S1).

Strain RerS4 grew optimally at 28 °C and pH 7.0, with viable growth observed at temperatures ranging from 20 to 40 °C and pH values of 4.0–10.0. NaCl tolerance assays revealed that the strain could survive in media containing 0–2% (*w*/*v*) NaCl, with maximum growth observed in salt-free conditions. Morphological observations showed that RerS4 developed well-branched, smooth, aseptate aerial hyphae with a width of 0.5–1.0 μm. Upon maturation on ISP 3 medium, the aerial mycelia formed smooth-surfaced spore chains composed of cylindrical spores measuring 1.0–2.0 μm × 0.5–1.0 μm (Figure 1B). Physiologically, strain RerS4 was positive for siderophore, amylase, catalase, and urease production, while negative for Tween 20/40/80 hydrolysis, cellulose decomposition, H_2_S production, and nitrate reduction. The strain utilized a broad spectrum of carbon and nitrogen sources. Differential phenotypic characteristics between RerS4 and its closely related *Streptomyces* species are summarized in Table S2.

**Figure 2:**
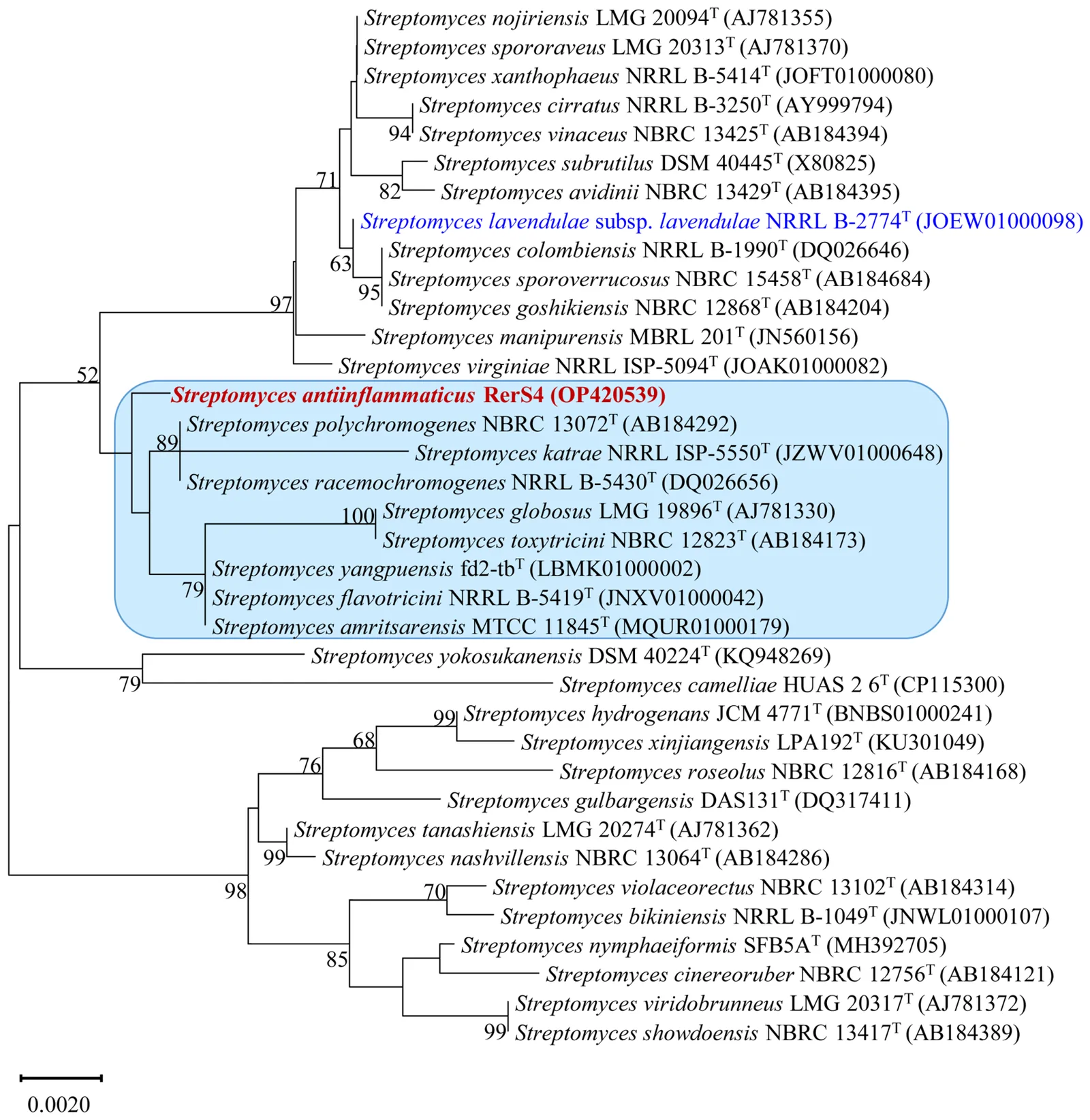
Neighbor-joining tree based on 16S rRNA gene sequences showing the relationship between strain RerS4 (1523 bp) and related species of the genus *Streptomyces*. Bootstrap percentages (≥50%) based on 1000 replications are listed at the nodes. Bar, 0.0020 substitutions per nucleotide position. Strain marked in blue: strain did not cluster with RerS4 in the 16S rRNA phylogenetic tree but shared the same branch in the whole-genome tree and was subjected to ANI and dDDH analyses; Strain marked in red: the isolate to be identified in this study; strains in the blue box: strains for ANI and dDDH analyses.

**Figure 3:**
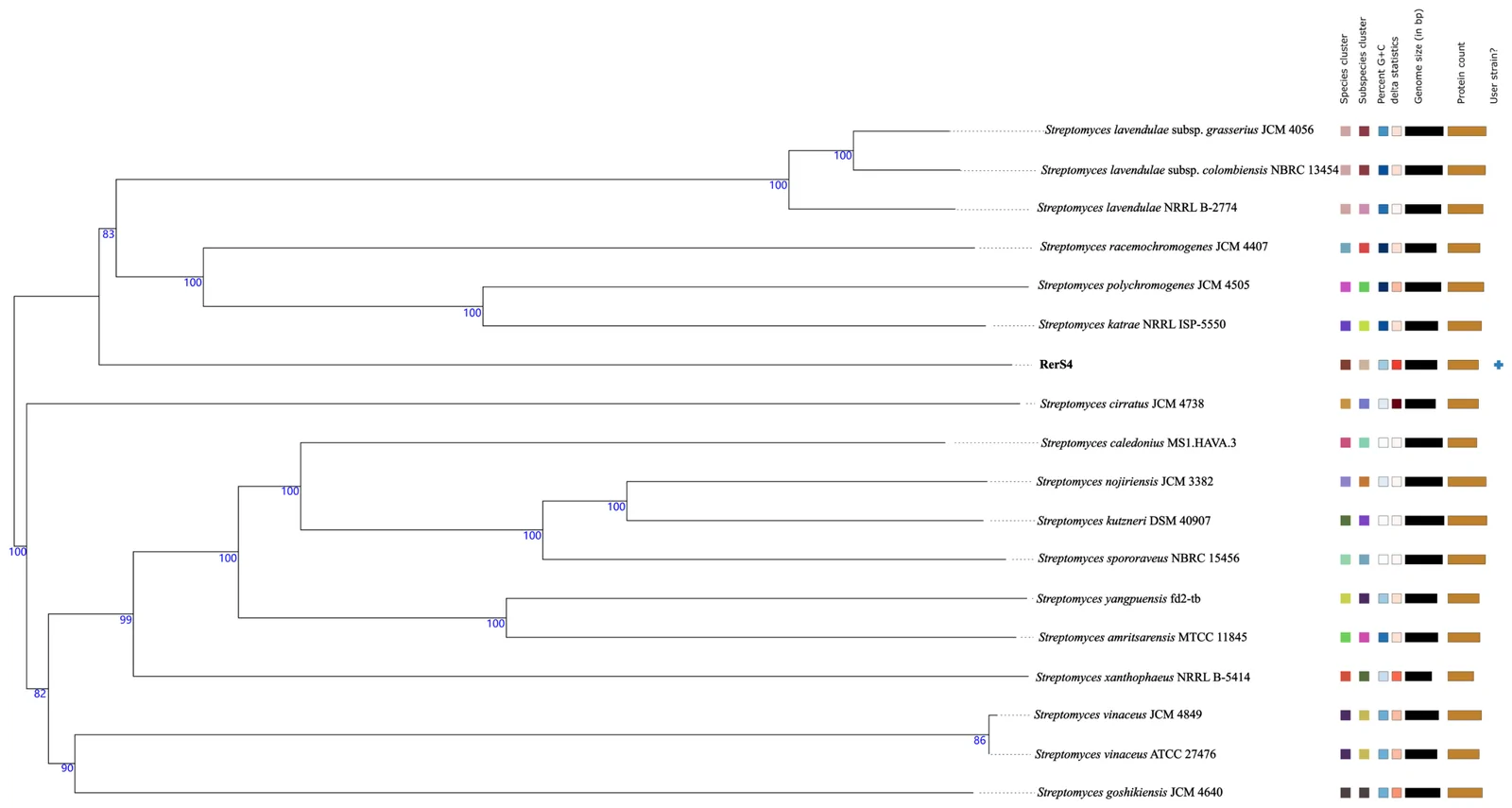
Whole-genome sequence tree generated with TYGS for strains RerS4 and closely related species of genus *Streptomyces*. Tree inferred with FastME from GBDP distances calculated from genome sequences. Branch lengths are scaled in terms of GBDP distance formula d5; numbers above branches are GBDP pseudo-bootstrap support values from replicates.

The full-length 16S rRNA gene sequence of strain RerS4 (1523 bp; GenBank accession: OP420539) exhibited the highest sequence similarity (99.72%) to *Streptomyces racemochromogenes* NRRL B-5430^T^ and *Streptomyces polychromogenes* NBRC 13072^T^. The 16S rRNA-based phylogenetic tree showed that strain RerS4 formed an independent clade closely clustered with *S. polychromogenes* NBRC 13072^T^, *Streptomyces katrae* NRRL ISP-5550^T^, and *S. racemochromogenes* NRRL B-5430^T^ (Figure 2). Whole-genome phylogenetic analysis further indicated that RerS4 grouped within a large clade comprising *Streptomyces lavendulae* subsp. *grasserius* JCM 4056, *Streptomyces lavendulae* subsp. *colombiensis* NBRC 13454, *Streptomyces lavendulae* subsp. *lavendulae* NRRL B-2774^T^, *S. racemochromogenes* NRRL B-5430^T^, *S. polychromogenes* NBRC 13072^T^, and *S. katrae* NBRC 13447^T^ (Figure 3). Notably, *S. lavendulae* subsp. *lavendulae* NRRL B-2774^T^ did not cluster with RerS4 in the 16S rRNA phylogenetic tree but shared the same branch in the whole-genome tree. Accordingly, ANI and dDDH analyses were performed for these closely related *Streptomyces* strains. The results demonstrated that both dDDH values (<70%) and ANI values (<95%) between RerS4 and all reference strains were below the standard thresholds for bacterial species delineation [17,18] (Table 1). Combined with the distinct morphological and physiological differences between RerS4 and its phylogenetic relatives (Tables S1 and S2), this genomic and phenotypic evidence collectively confirms that strain RerS4 represents a novel *Streptomyces* species, for which the name *Streptomyces antiinflammaticus* is proposed.

### 2.2. Fermentation Medium Screening for Anti-Inflammatory Active Compound Production

**Figure 4:**
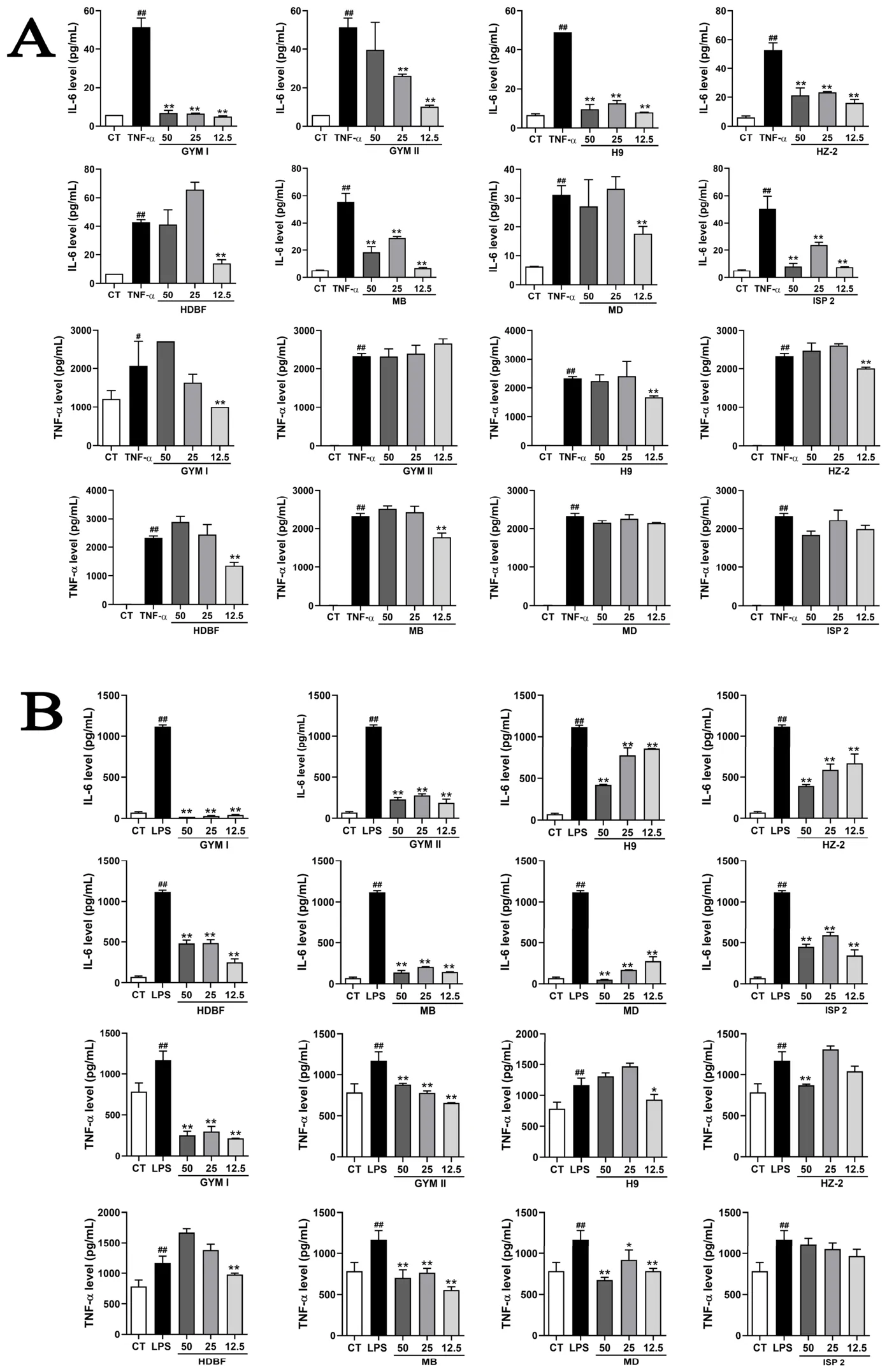
Effects of eight fermentation broths at different concentrations on IL-6 and TNF-α levels in (**A**) A549 alveolar epithelial cells and (**B**) THP-1 monocyte/macrophages. Cells were stimulated with LPS and TNF-α in the presence or absence of fermentation broths, and IL-6 and TNF-α secretion was measured by ELISA. Data are expressed as mean ± SD (*n* = 3). #: *p* < 0.05 vs. control group; ##: *p* < 0.01 vs. control group; *: *p* < 0.05 vs. LPS or TNF-α treated group; **: *p* < 0.01 vs. LPS or TNF-α-treated group.

The anti-inflammatory activities of fermentation broths from eight different media were systematically evaluated using TNF-α-stimulated A549 cells and LPS-stimulated THP-1 macrophages by detecting the secretion of pro-inflammatory cytokines IL-6 and TNF-α (Figure 4). In A549 cells, high, medium, and low concentrations (50, 25, and 12.5 µg/mL) of GYM I, H9, HZ-2, MB, and ISP 2 fermentation broths, as well as low concentrations of GYM II, HDBF, and MD broths, significantly suppressed IL-6 secretion. Meanwhile, 12.5 µg/mL GYM I, H9, HZ-2, HDBF, and MB broths markedly inhibited TNF-α release. These results indicated that H9, GYM I, and HZ-2 media were optimal for yielding metabolites with potent anti-inflammatory activity in A549 cells (Figure 4A).

In THP-1 macrophages, all eight fermentation broths exerted varying degrees of inhibitory effects on IL-6 secretion across the tested concentrations. Specifically, GYM I, GYM II, MB, and MD broths significantly reduced IL-6 secretion at all three concentrations. Additionally, all concentrations of GYM I, GYM II and MB broths exhibited obvious TNF-α inhibitory activity (Figure 4B). Except for ISP 2 broth, all other seven media effectively alleviated inflammatory responses in THP-1 cells. Based on the comprehensive anti-inflammatory performance in both cell models, GYM I medium was ultimately selected for large-scale fermentation and subsequent compound isolation and purification.

### 2.3. Structure Elucidation

**Figure 5:**
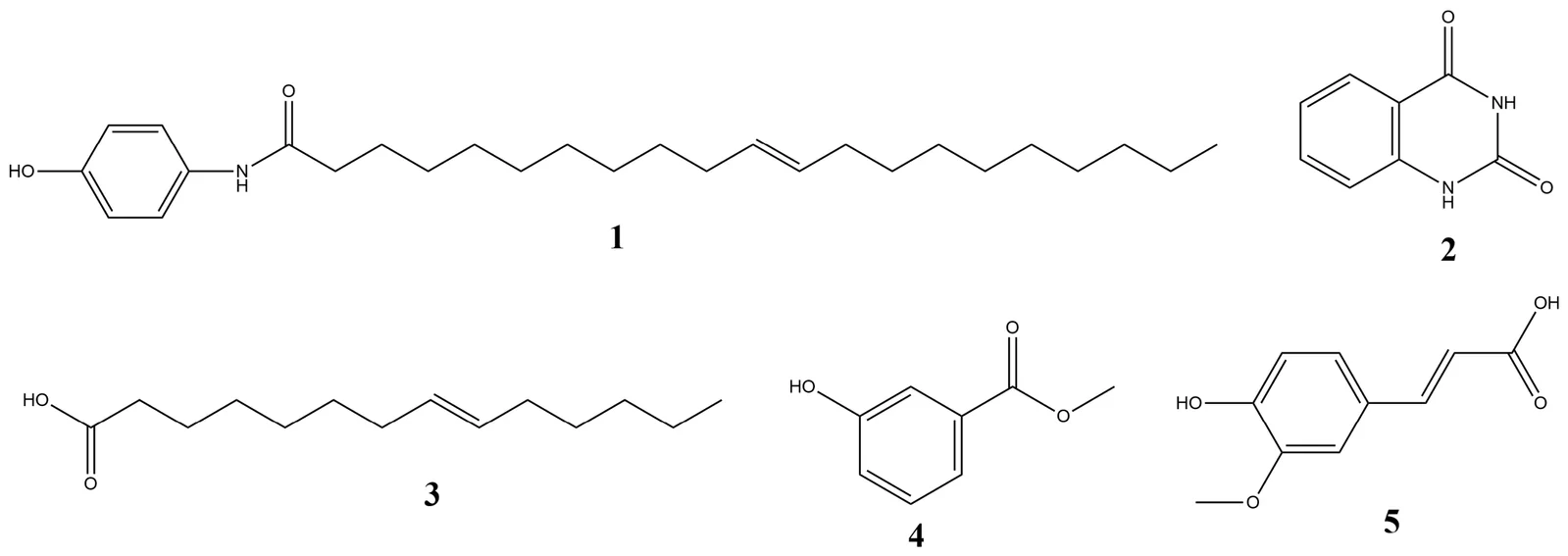
Chemical structures of compounds **1**–**5** isolated from RerS4.

Five secondary metabolites were isolated and purified from the GYM I medium fermentation broth of strain *S. antiinflammaticus* RerS4 (Figure 5). The structural characteristics of each compound were elucidated via HR-ESI-MS and comprehensive NMR spectroscopic analysis.

**Figure 6:**
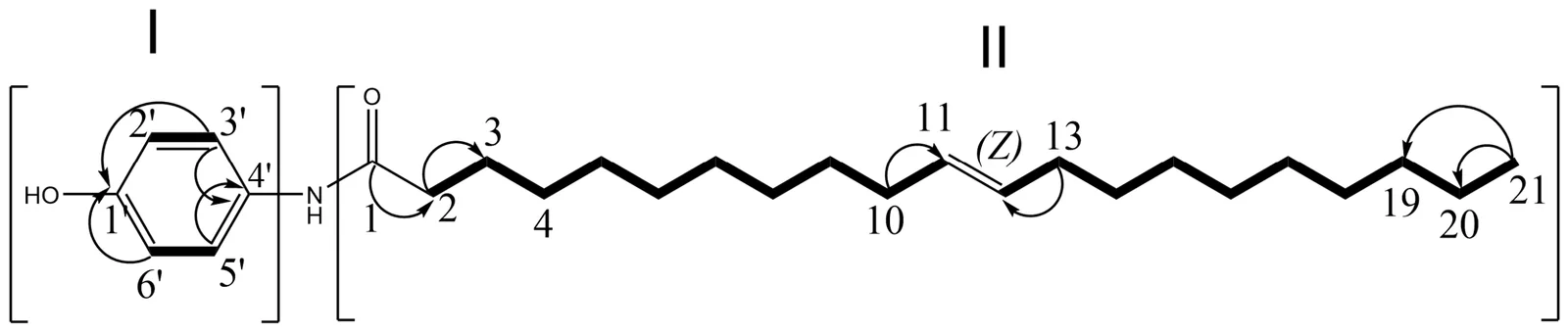
2D nuclear magnetic resonance (NMR) correlations of compound **1**. Bold solid lines: ^1^H-^1^H COSY; arrows: HMBC correlations.

Compound **1** was obtained as a yellowish powder. Its HR-ESI-MS spectrum exhibited a [M + K]^+^ ion peak at *m*/*z* 454.3455 (calcd. 454.3450), corresponding to the molecular formula C_27_H_45_NO_2_ (Figures S1 and S2). The 1D and 2D NMR spectroscopic data were fully analyzed to establish its planar structure (Figures S3–S7). The ^1^H NMR (600 MHz, Methanol-d4) spectrum showed four aromatic protons as two doublets at δ_H_ 7.33 (2H, d, *J* = 8.7 Hz, H-2′, 6′) and 6.76 (2H, d, *J* = 8.7 Hz, H-3′, 5′), two olefinic methines at δ_H_ 5.34 (2H, dd, *J* = 10.0, 6.9 Hz, H-11, 12), and aliphatic signals: an amide α-methylene at δ_H_ 2.19 (2H, t, *J* = 7.4 Hz, H-2), a β-methylene at 1.60 (2H, t, *J* = 7.4 Hz, H-3), allylic methylenes at 2.03 (4H, q, *J* = 6.9 Hz, H-10, 13), aliphatic methylenes at 1.34–1.29 (26H, m, H-4–9, H-14–20), a terminal methyl at 0.90 (3H, t, *J* = 7.0 Hz, H-21), and an exchangeable amide proton at 8.50 (Figure S3). The ^1^H and ^13^C NMR spectra collectively exhibited 27 carbon resonances: a para-substituted benzene ring (δ_C_ 136.7 C-1′, 160.5 C-4′, 132.6 C-2′,6′, 116.4 C-3′,5′), 17 methylene carbons (δ_C_ 36.5 C-2, 33.1, 30.3–30.8, 28.1, 26.9 C-10/13, 23.7, etc.), two olefinic methine carbons (δ_C_ 130.8 C-11, C-12), one methyl carbon (δ_C_ 14.4 C-21), and one amide carbonyl (δ_C_ 179.6 C-1) (Table 2 and Figures S5 and S6). The ^1^H–^1^H COSY (Figure S6) revealed spin systems for H-2′/H-3′/H-5′/H-6′ (fragment I) and H-2–H-4, H-9–H-14, H-19–H-21 (fragment II, Figure 6). HMBC (Figure S7) from H-2′, 6′→C-1′, H-3′, 5′→C-4′, H-2→C-1/C-3, H-10,13→C-11,12, H-21→C-20,19 secured connectivity. C-1′ (aryl), C-1 (carbonyl) and C-2 indicated an aryl amide link. The coupling constant (*J* = 10.0 Hz) of the olefinic protons indicated a *cis* configuration of the Δ double bond, akin to 9(*Z*)-octadecenamide [19] and (9*Z*)-*N*-(4-Hydroxyphenyl)-9-octadecenamide [20]. Comparison with SciFinder indicates that while the main scaffold is known, the long fatty acyl chain attached via the amide bond (differing in the number of carbon atoms) confirms compound **1** as a novel compound (Figure S8). Accordingly, compound **1** was identified as (*Z*)-*N*-(4-hydroxyphenyl)henicos-11-enamide (Figure 6).

Compound **2** was obtained as a brown powder. Its HR-ESI-MS data showed a [M + H]^+^ peak at *m*/*z* 163.0449, matching the molecular formula C_8_H_6_N_2_O_2_. ^1^H-NMR (600 MHz, CD_3_OD) δ: 8.01 (1H, dd, *J* = 7.9, 1.3 Hz, H-5), 7.65 (1H, m, H-6), 7.24 (1H, t, *J* = 7.5 Hz, H-7), 7.18 (1H, s, H-8); ^13^C-NMR (150.9 MHz, CD_3_OD) δ: 152.6 (C-2), 165.3 (C-4), 115.9 (C-4a), 128.4 (C-5), 136.5 (C-6), 124.2 (C-7), 116.5 (C-8), 142.3 (C-8a). The above spectral data were in good agreement with those reported in the literature [21], leading to the identification of compound **2** as 2,4-quinazolinedione.

Compound **3** was obtained as a white powder. The HR-ESI-MS [M + H]^+^ ion at *m*/*z* 227.1937 corresponded to the molecular formula C_14_H_26_O_2_. ^1^H-NMR (600 MHz, CD_3_OD) δ: 5.35 (2H, t, *J* = 4.9, H-7, H-8), 2.04 (2H, m, H-13), 1.6 (2H, m, H-2), 1.15–1.48 (18H, m), 0.9 (3H, t, *J* = 6.8, H-14); ^13^C-NMR (150.9 MHz, CD_3_OD) δ: 174.6 (C = O, C-1), 32.9 (C-2), 26.1 (C-3), 28.2 (C-4), 30.2 (C-5), 30.3 (C-6), 30.9 (C-7), 130.9 (C-8), 130.8 (C-9), 30.0 (C-10), 23.8 (C-11), 28.1 (C-12), 14.3 (C-13). The above spectral data were in good agreement with those reported in the literature [22], leading to the identification of compound **3** as (*E*)-tetradec-8-enoic acid.

Compound **4** was obtained as a white powder. Its HR-ESI-MS exhibited a [M + Na]^+^ peak at *m*/*z* 175.0449, consistent with the molecular formula C_8_H_8_O_3_. ^1^H-NMR (600 MHz, CD_3_OD) δ: 7.47 (1H, d, *J* = 7.7 Hz, H-2), 7.41 (1H, t, *J* = 2.0 Hz, H-3), 7.28 (1H, t, *J* = 7.9 Hz, H-6), 7.01 (1H, m, H-5), 3.88 (3H, s, CH3); ^13^C-NMR (150.9 MHz, CD_3_OD) δ: 168.6 (C-1), 132.6 (C-2), 117.0 (C-3), 158.8 (C-4), 121.2 (C-5), 130.6 (C-6), 121.6 (C-7), 52.6 (-OCH_3_). The above spectral data were in good agreement with those reported in the literature [23], leading to the identification of compound **4** as methyl-3-hydroxybenzoate.

Compound **5** was obtained as a light brown powder. The HR-ESI-MS [M + H]^+^ ion at *m*/*z* 195.0536 corresponded to the molecular formula C_10_H_10_O_4_ (Figures S9 and S10). ^1^H NMR (600 MHz, DMSO-d_6_) δ: 7.49 (1H, d, *J* = 15.9 Hz, H-7), 7.27 (1H, s, H-2), 7.07 (1H, d, *J* = 8.1 Hz, H-6), 6.76 (1H, d, *J* = 8.1 Hz, H-5), 6.36 (1H, d, *J* = 15.9 Hz, H-8); ^13^C-NMR (150.9 MHz, DMSO-d_6_) δ: 168.0 (C-9), 149.0 (C-4), 147.9 (C-3), 144.4 (C-7), 125.8 (C-1), 122.8 (C-6), 115.7 (C-8), 115.5 (C-5), 111.1 (C-2), 55.7 (-OCH_3_) (Figures S11–S15). The spectral data were consistent with those reported in the literature [24], and the compound was identified as 3-methoxy-4-hydroxycinnamic acid, namely ferulic acid.

### 2.4. Anti-Inflammatory Mechanism of Compounds **1** and **5**

CCK-8 cytotoxicity assays verified that all five compounds exhibited no obvious cytotoxicity against A549 and THP-1 cells at concentrations up to 200 μM after 24 h treatment, with cell viability maintained above 90% in all treatment groups (Figure S16). This result indicated that the inhibitory effects of the tested compounds on inflammatory cytokine secretion were independent of cytotoxic damage. Therefore, concentrations of 100, 150, and 200 μM were selected for subsequent mechanism exploration.

**Figure 7:**
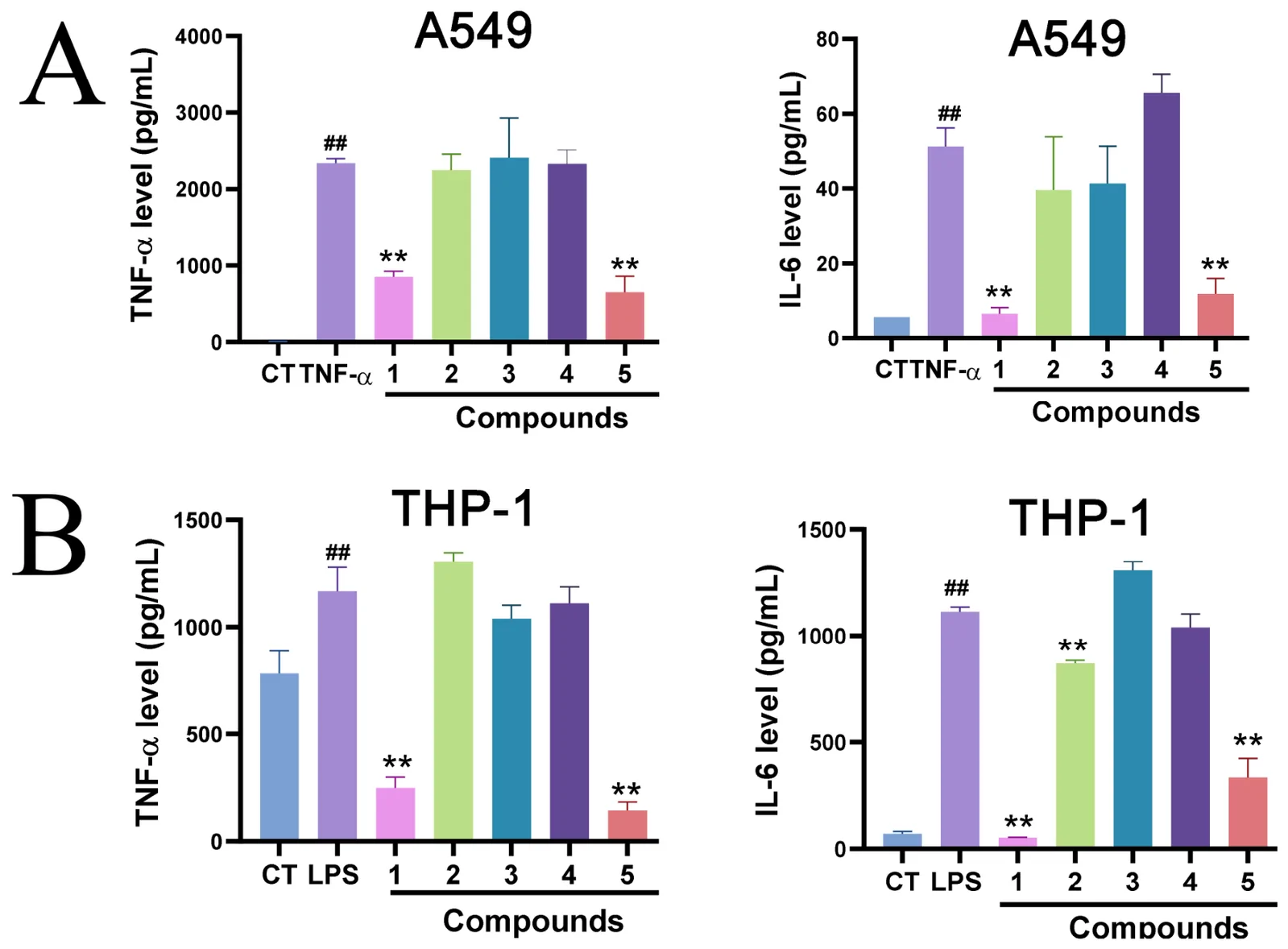
Effects of five compounds (**1**–**5**) on IL-6 and TNF-α levels in (**A**) A549 alveolar epithelial cells and (**B**) THP-1 monocyte/macrophages. Cells were stimulated with LPS or TNF-α in the presence or absence of compounds, and IL-6 and TNF-α secretion was measured by ELISA. Data are expressed as mean ± SD (*n* = 3). ##: *p* < 0.01 vs. control group; **: *p* < 0.01 vs. LPS- or TNF-α-treated group.

ELISA results demonstrated that both compounds **1** and **5** significantly suppressed the secretion of pro-inflammatory TNF-α and IL-6 in TNF-α-stimulated A549 cells and LPS-stimulated THP-1 macrophages (Figure 7). To further clarify the underlying molecular mechanisms, qRT-PCR and Western blotting analyses were performed to detect inflammatory gene expression and key signaling pathway activation.

**Figure 8:**
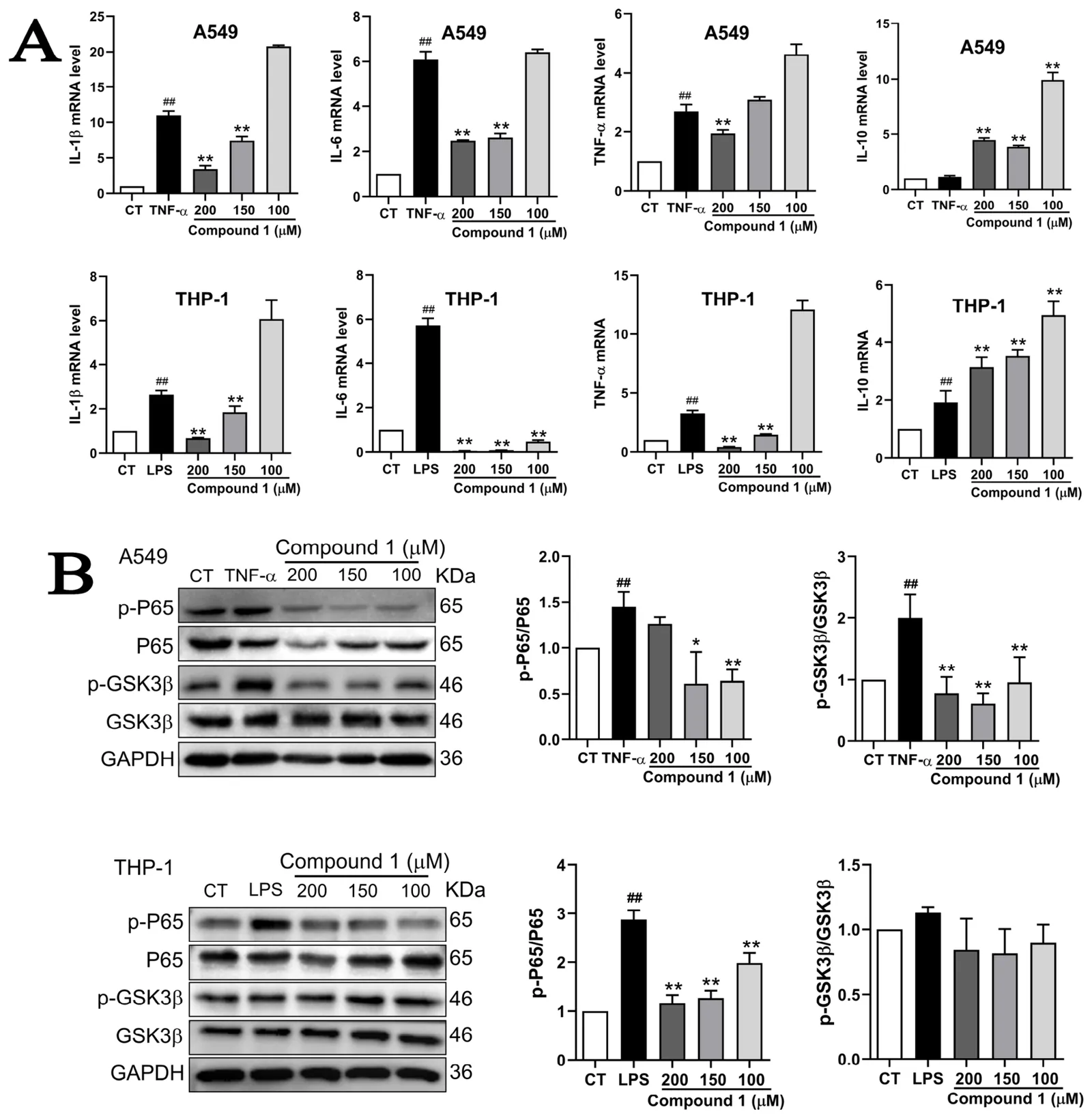
Compound **1** suppresses pro-inflammatory cytokine mRNA expression and GSK3β/NF-κB signaling in A549 and THP-1 cells. (**A**) Effects of compound **1** at different concentrations on the mRNA levels of IL-1β, IL-6, TNF-α and IL-10 in A549 alveolar epithelial cells and THP-1 monocyte/macrophages. Cells were stimulated with LPS or TNF-α in the presence or absence of compound **1**, and mRNA expression was analyzed by qPCR. (**B**) Effects of compound **1** on GSK3β/NF-κB pathway protein expression in A549 and THP-1 cells. Cells were stimulated with LPS in the presence or absence of compound 1, and protein levels of p-p65 (Ser536), p65, p-GSK3β (Tyr216), and GSK3β were analyzed by Western blot. Data are expressed as mean ± SD (*n* = 3). ##: *p* < 0.01 vs. control group; *: *p* < 0.05 vs. LPS- or TNF-α-treated group; **: *p* < 0.01 vs. LPS- or TNF-α-treated group.

For compound **1**, qRT-PCR results showed that 200 μM compound **1** significantly downregulated the mRNA expression of pro-inflammatory cytokines (IL-1β, IL-6, TNF-α) and upregulated the anti-inflammatory cytokine IL-10 in both A549 and THP-1 cells. At 150 μM, compound **1** also inhibited pro-inflammatory gene expression in THP-1 cells and induced IL-10 expression in both cell lines (Figure 8A). Western blotting analysis revealed that compound **1** concentration-dependently suppressed the phosphorylation of p65 (Ser536) in A549 (100–200 μM) and THP-1 cells (100–200 μM). By contrast, compound **1** inhibited GSK3β (Tyr216) phosphorylation only in A549 cells, with no significant effect observed in THP-1 macrophages (Figure 8B). The original Western blot films are shown in Supplementary File S2.

**Figure 9:**
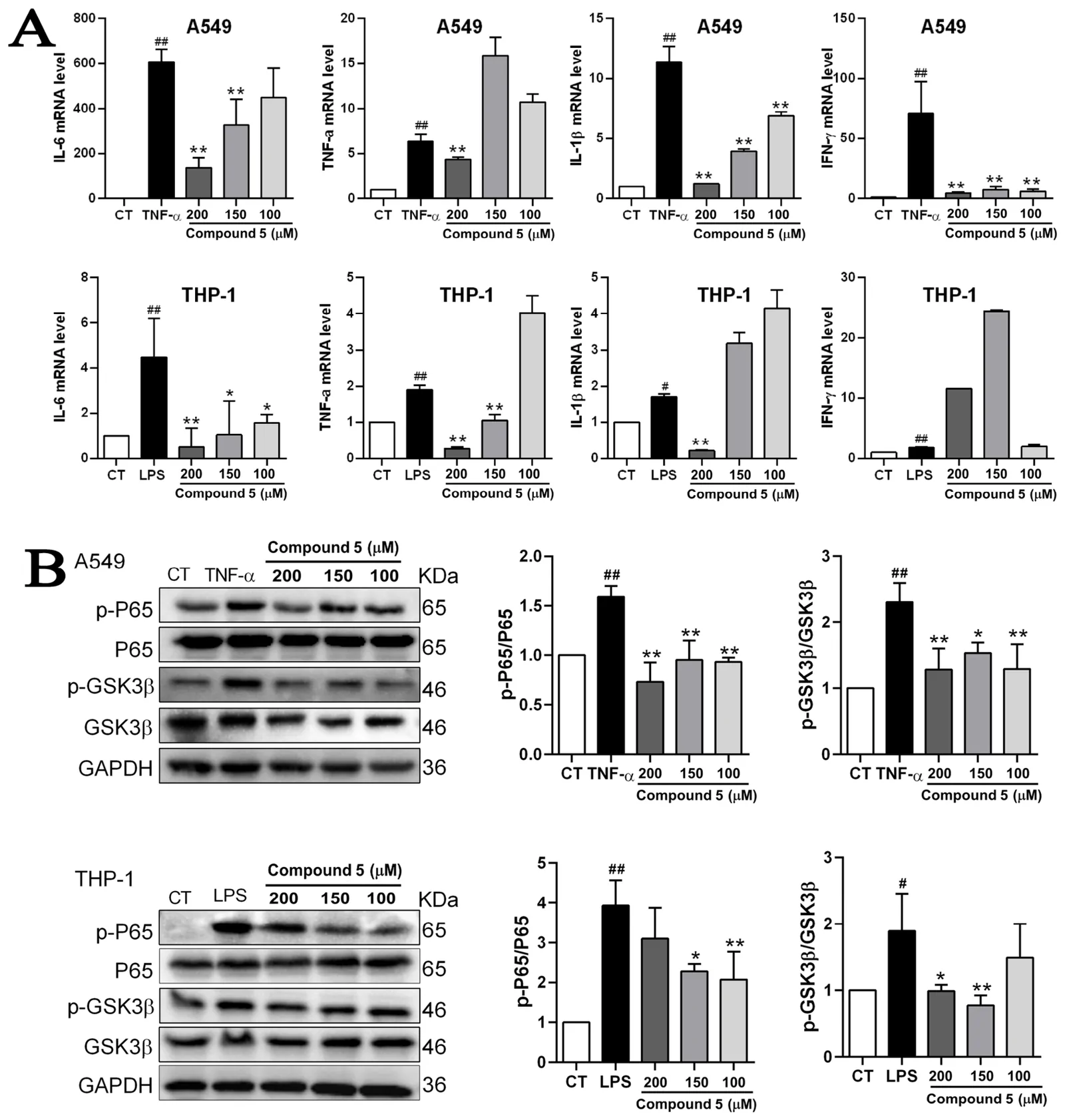
Compound **5** suppresses pro-inflammatory cytokine mRNA expression and GSK3β/NF-κB signaling in A549 and THP-1 cells. (**A**) Effects of compound **5** at different concentrations on the mRNA levels of IL-1β, IL-6, TNF-α and IL-10 in A549 alveolar epithelial cells and THP-1 monocyte/macrophages. Cells were stimulated with LPS or TNF-α in the presence or absence of compound **5**, and mRNA expression was analyzed by qPCR. (**B**) Effects of compound **5** on GSK3β/NF-κB pathway protein expression in A549 and THP-1 cells. Cells were stimulated with LPS or TNF-α in the presence or absence of compound **5**, and protein levels of p-p65 (Ser536), p65, p-GSK3β (Tyr216), and GSK3β were analyzed by Western blot. Data are expressed as mean ± SD (*n* = 3). #: *p* < 0.05 vs. control group; ##: *p* < 0.01 vs. control group; *: *p* < 0.05 vs. LPS- or TNF-α-treated group; **: *p* < 0.01 vs. LPS- or TNF-α-treated group.

For compound **5**, 200 μM treatment markedly reduced the mRNA levels of IL-1β, IL-6, and TNF-α in both cell lines. At 150 μM, compound **5** inhibited the expression of IL-6, IL-1β, and IFN-γ in A549 cells, and suppressed IL-6 and TNF-α expression in THP-1 cells (Figure 9A). Western blotting results confirmed that compound **5** concentration-dependently inhibited the phosphorylation of both p65 (Ser536) and GSK3β (Tyr216) in A549 cells at 100–200 μM. In THP-1 cells, 150 μM compound **5** significantly reduced the phosphorylation levels of both signaling proteins (Figure 9B). The original Western blot films are shown in Supplementary File S2.

## 3. Discussion

In recent years, prokaryotic taxonomy has undergone a fundamental paradigm shift from traditional culture-dependent polyphasic characterization to genome-based classification. Among currently available genomic metrics, dDDH and ANI have been widely recognized as gold-standard criteria for bacterial species delineation [12,25]. In the present study, the isolate RerS4 exhibited dDDH values of 26.7–31.9% and ANI values of 82.87–85.87% relative to its closest phylogenetic relatives. These values are distinctly below the generally accepted species thresholds of 70% for dDDH and 95–96% for ANI (Table 1), thereby supporting the taxonomic assignment of strain RerS4 as a novel species within the genus *Streptomyces*. Consistent with genomic evidence, obvious phenotypic discrepancies were also observed between RerS4 and its closely related reference strains (Tables S1 and S2). For example, on ISP 7 medium, the substrate mycelium of RerS4 is dark brown, while the closely related strains exhibit beige, cream, or brownish orange substrate mycelia [26] (Table S2). Collectively, these genomic and phenotypic results confirm that strain RerS4 represents a novel *Streptomyces* species, for which the name *Streptomyces antiinflammaticus* is proposed.

The genus *Streptomyces* is well known as a prolific reservoir of bioactive natural products with pharmaceutical significance, including anti-inflammatory compounds, immunomodulators, and clinically approved anti-infective agents [27,28,29]. Accumulating evidence indicates that taxonomic novelty frequently corresponds to unique biosynthetic potential and specialized metabolic diversity [14,30]. Our previous genomic analysis revealed that strain RerS4 possesses a rich and phylogenetically distinct repertoire of biosynthetic gene clusters (BGCs). Notably, multiple uncharacterized gene sequences associated with non-ribosomal peptide synthetase (NRPS) and polyketide synthase (PKS) pathways, as well as other specialized metabolic loci, were identified in the RerS4 genome [15]. Strain RerS4 was isolated from the rhizosphere of *R. glutinosa*, a traditional medicinal plant renowned for its anti-inflammatory and heat-clearing pharmacological properties [31,32,33]. The well-documented anti-inflammatory efficacy of *R. glutinosa* suggests that its rhizosphere-associated actinomycetes may produce immunomodulatory secondary metabolites that synergize or complement the bioactive constituents of the host plant [34,35].

Nevertheless, the translation of genomic biosynthetic potential into actual chemical products is highly dependent on microbial cultivation conditions. The expression of secondary metabolic BGCs is tightly regulated by environmental cues, and different fermentation media can selectively activate or silence specific biosynthetic pathways [36]. To unlock the latent metabolic potential of strain RerS4, we performed a systematic fermentation medium screening. Under optimized culture conditions, five secondary metabolites, including one previously undescribed compound, were successfully isolated and purified from the fermentation broth of RerS4.

Among the five isolated metabolites, compounds **1** and **5** exhibited prominent anti-inflammatory activities, as evidenced by their significant suppression of the pro-inflammatory cytokines TNF-α, IL-6, and IL-1β in both A549 epithelial cells and THP-1 macrophage cells. This bioactivity validates the hypothesis that actinomycetes associated with medicinal plants such as *R. glutinosa* serve as promising resources for novel immunomodulatory metabolites [37,38]. Importantly, compound **1** uniquely upregulated the anti-inflammatory cytokine IL-10 at both 150 μM and 200 μM, demonstrating a dual immunomodulatory function: it simultaneously inhibits pro-inflammatory signaling and promotes inflammatory resolution. This bidirectional regulatory property is particularly valuable for therapeutic development, as small molecules capable of suppressing excessive inflammation while enhancing anti-inflammatory feedback loops are highly desirable for the treatment of chronic inflammatory disorders [39,40].

Mechanistically, both compounds **1** and **5** exert anti-inflammatory effects by targeting the NF-κB signaling pathway, as indicated by the pronounced inhibition of p65 phosphorylation at the Ser536 residue in both A549 and THP-1 cell lines (Figure 8 and Figure 9). However, the two compounds exhibit distinct regulatory effects on the GSK3β signaling node. Compound **1** suppressed GSK3β phosphorylation at Tyr216 in A549 cells but showed no significant effect in THP-1 cells, implying that its anti-inflammatory activity in macrophages may be independent of GSK3β or mediated through alternative signaling cascades, such as the MAPK or Nrf2 pathways [41,42]. In contrast, compound **5** inhibited GSK3β phosphorylation in both cell types, albeit with distinct dose-dependent responses (Figure 9). These findings reveal cell-type and compound-specific differences in GSK3β involvement, which may stem from intrinsic variations in basal GSK3β activity and upstream kinase network regulation between alveolar epithelial cells and macrophages. Furthermore, compound **5** displayed a broader regulatory spectrum than compound **1**, as it additionally reduced IFN-γ mRNA expression in A549 cells. As a signature cytokine of Th1-type adaptive immunity, IFN-γ is rarely targeted by microbial-derived small molecule anti-inflammatory agents [43]. This observation suggests that compound **5** may modulate both innate and adaptive immune responses, which warrants further validation using in vivo animal models or cell co-culture systems.

Several limitations of the present study should be acknowledged. First, the fermentation yields of compounds **1** and **5** were relatively low, which hinders further in vivo pharmacokinetic characterization and systematic therapeutic evaluation. Second, although CCK-8 cytotoxicity assays confirmed that the tested concentrations (up to 200 μM) of the two compounds did not impair cell viability, a more comprehensive cytotoxicity assessment across wider concentration gradients and multiple cell lines is required to fully validate their safety profiles. Third, all mechanistic conclusions in this study are derived from in vitro cellular models. Whether these compounds can inhibit NF-κB/GSK3β signaling and elevate IL-10 expression in animal models of inflammatory diseases (e.g., ulcerative colitis and rheumatoid arthritis) remains to be verified. In addition, the current results only demonstrate alterations in protein phosphorylation and gene transcription levels; direct molecular interactions between the two compounds and their putative targets (p65 and GSK3β) have not been experimentally confirmed. To address these limitations, future studies will focus on fermentation optimization to improve metabolite yields, expanded cytotoxicity evaluation, structural modification for structure–activity relationship analysis, direct target validation assays, and in vivo pharmacological verification. These efforts will lay a solid foundation for the translational development of these novel immunomodulatory natural products.

In conclusion, the present study taxonomically validates strain RerS4 as a novel *Streptomyces* species, designated *Streptomyces antiinflammaticus*, which was isolated from the rhizosphere of the medicinal plant *R. glutinosa*. Functional characterization confirmed this strain as a prolific source of structurally unique and biologically active anti-inflammatory metabolites. Five secondary metabolites were isolated from the optimized GYM I fermentation medium, among which the newly identified compound **1** and known compound **5** exert robust anti-inflammatory effects by modulating the GSK3β/NF-κB signaling axis in alveolar epithelial and macrophage cell models. Collectively, these findings broaden the taxonomic diversity of the genus *Streptomyces* and provide compelling evidence that phylogenetically distinct, medicinal plant-associated actinomycetes represent a valuable reservoir for the discovery of novel immunomodulatory natural products.

## 4. Materials and Methods

### 4.1. Microbial Strains and Culture Conditions

Strain RerS4 was isolated from the rhizosphere soil of ring rot-infected *R. glutinosa* [14]. The strain has been deposited in the China Center for Type Culture Collection (CCTCC NO. M 20251535; Figure S17) and preserved in the Biochemistry and Molecular Biology Research Section, School of Pharmacy, Henan University of Traditional Chinese Medicine. For routine cultivation, strain RerS4 was grown on ISP 3 medium at 28 °C for 7 days. The obtained spores were inoculated into ISP 2 medium and cultured for 2 days to prepare seed cultures, which were subsequently inoculated into eight different fermentation media (Table S3) at an inoculum volume of 6% for fermentation cultivation.

### 4.2. Taxonomic Characterization of Strain RerS4

The growth characteristics of strain RerS4 were evaluated on ISP 3 agar at temperatures ranging from 4 °C to 45 °C for 14 days. The tolerance to pH (pH 2–12) and NaCl concentration (0–10%, *w*/*v*) was determined in shaking ISP 2 broth at 28 °C for 14 days. A set of physiological and biochemical properties, including sole carbon and nitrogen source utilization, milk reaction, cellulose decomposition, nitrate reduction, gelatin liquefaction, and the production of H_2_S, protease, Tween-hydrolyzing enzyme, and urease, were tested according to previously described methods [44]. Genome-based taxonomic identification was performed using digital DNA-DNA hybridization (dDDH) [45] and average nucleotide identity (ANI) analyses via the JspeciesWS platform [46]. Colonial morphology was observed on multiple media (ISP series, Bennett’s medium, and nutrient agar) after 14 days of incubation at 28 °C, while spore surface morphology was visualized using a scanning electron microscope (Hitachi High-Technologies Corporation, Hitachinaka, Japan, model SU8010).

### 4.3. Preparation of Fermentation Broth Extracts

Strain RerS4 was cultured on ISP 3 agar slants and activated on fresh ISP 3 plates at 28 °C for 7 days. A well-sporulated agar block was used to prepare a spore suspension in sterile water, adjusted to 1.0 × 10^7^ CFU/mL. This standardized spore suspension was inoculated (5%, *v*/*v*) into 50 mL of ISP 2 seed medium in 250 mL Erlenmeyer flasks and incubated on a rotary shaker at 220 rpm and 28 °C for 48 h, until the culture reached mid-exponential phase (OD_450_ = 0.6–0.8). The seed culture was then inoculated at a fixed 6% (*v*/*v*) into each of eight tested media (listed in Table S3) dispensed as 50 mL aliquots in 250 mL Erlenmeyer flasks. Three independent biological replicates were prepared per medium, and all flasks were incubated at 28 °C and 220 rpm for 7 days.

After incubation, the whole broth of each replicate was centrifuged at 12,000× *g* for 10 min to separate the supernatant from the mycelial pellet. The supernatant was extracted three times with an equal volume of ethyl acetate; the combined organic phases were concentrated under reduced pressure to afford the crude extract. The crude extract was dissolved in cell-culture-grade DMSO at 100 mg/mL, filter-sterilized through a 0.22 μm membrane, and stored at −20 °C prior to bioactivity screening.

### 4.4. Fermentation, Extraction, and Isolation of Compounds

On the basis of anti-inflammatory activity in the small-scale screening, GYM I medium was selected for scale-up. Large-scale fermentation was performed in 200 × 1 L Erlenmeyer flasks, each containing 250 mL GYM I medium, inoculated at 6% (*v*/*v*) with the same standardized 48 h seed culture as above, incubated at 28 °C and 220 rpm for 7 days. The extraction procedure was identical in chemistry to the small-scale protocol. The total extract (36 g) was thoroughly fractionated using medium-pressure liquid chromatography (MPLC) with a methanol–water gradient (*v*/*v* 20:80, 40:60, 60:40, 80:20, 100:0, each 3000 mL), yielding five fractions (**a**–**e**). Fraction **b** (5.4 g) was mixed with silica gel at a 1:1 ratio, loaded by dry packing, and subjected to silica gel column chromatography. Elution was performed with petroleum ether/ethyl acetate (1:1, *v*/*v*) to obtain **b1**–**b8**. Subfraction **b2** (56 mg) was further purified by Sephadex LH-20 (MeOH), affording five minor parts (**b2a**–**b2e**), and then **b2a** (11 mg) was separated by prep-HPLC using a linear elution (MeCN/H_2_O, 90:10, *v*/*v*, 25 min, 3 mL·min^−1^) to yield compound **1** (2.3 mg, t*_R_* = 19.2 min). Compound **2** (2.5 mg, MeCN/H_2_O, from 60/40 to 90/10, *v*/*v*, 30 min, 3 mL·min^−1^, t*_R_* = 10.3 min) and compound **3** (2.7 mg, MeCN/H_2_O, from 40/60 to 90/10, *v*/*v*, 30 min, 3 mL·min^−1^, t*_R_* = 15.7 min) were obtained from subfraction **b2b** via prep-HPLC. Compound **4** (3.0 mg, MeCN/H_2_O, from 15/85 to 30/70, *v*/*v*, 30 min, 3 mL·min^−1^, t*_R_* = 19.8 min) and compound **5** (2.3 mg, MeCN/H_2_O, from 12/88 to 30/70, *v*/*v*, 25 min, 3 mL·min^−1^, t*_R_* = 27.8 min) were isolated from fractions **b2c** using semi-preparative HPLC.

NMR spectra were recorded on a Bruker Avance III 600 MHz spectrometer (Bruker Corporation, Karlsruhe, Germany) at 298 K. ^1^H and ^13^C NMR, as well as 2D experiments including COSY, HSQC, and HMBC, were acquired in CD_3_OD or DMSO-d_6_; chemical shifts (δ) are reported in ppm relative to the residual solvent signal δ_H_ 3.31 or δ_C_ 49.0 for CD_3_OD and δ_H_ 2.50 or δ_C_ 39.52 for DMSO-d_6_ as internal references. The spectrometer operated at 600 MHz for ^1^H and 150.9 MHz for ^13^C. Data processing was performed using TopSpin (version 3.6, Bruker). High-resolution electron impact mass spectra (HREIMS) were obtained on a Waters, Manchester, UK, AutoSpec Premier P776 mass spectrometer.

### 4.5. Cell Lines and Culture Conditions

Human lung adenocarcinoma A549 cells and human acute monocytic leukemia THP-1 cells were purchased from the Cell Bank of Type Culture Collection of the Chinese Academy of Sciences (Shanghai, China). A549 cells were cultured in complete medium supplemented with 10% fetal bovine serum (FBS) and 1% penicillin–streptomycin mixture. THP-1 cells were maintained in RPMI 1640 medium containing 10% FBS. All cells were incubated at 37 °C in a humidified incubator with 5% CO_2_. THP-1 monocytes were differentiated into mature macrophages by stimulation with 100 ng/mL phorbol 12-myristate 13-acetate (PMA) for 48 h before subsequent experiments.

### 4.6. Cell Viability Assay (CCK-8)

To exclude cytotoxic interference with anti-inflammatory evaluation, cell viability was determined using the CCK-8 assay. A549 and THP-1 cells were seeded into 96-well plates at densities of 5 × 10^3^ and 1 × 10^4^ cells/well, respectively, and incubated overnight for adherence. Cells were then treated with compounds **1**–**5** at gradient concentrations of 0, 50, 100, and 200 μM for 24 h. After treatment, 10 μL of CCK-8 reagent (Dojindo, Kumanoto, Japan) was added to each well, and the plates were incubated at 37 °C for 4 h. The absorbance at 450 nm was measured using a microplate reader. Cell viability was calculated relative to the untreated control group. All experiments were performed in triplicate, and data were presented as mean ± standard deviation (SD).

### 4.7. ELISA

Inflammatory cell models were established via different stimuli: A549 cells were induced with 20 ng/mL TNF-α, while THP-1-derived macrophages were stimulated with 1 μg/mL LPS. After 24 h of intervention with the tested compounds, cell culture supernatants were collected. The concentrations of pro-inflammatory cytokines IL-6 and TNF-α in the supernatants were quantified using R&D Systems DuoSet ELISA kits (IL-6, cat. no. DY206-5; TNF-α, cat. no. DY210-5; Minneapolis, MN, USA) in accordance with the manufacturer’s standard protocols.

### 4.8. Quantitative Real-Time PCR

Total cellular RNA was extracted using Trizol reagent, and the concentration and purity of RNA were determined prior to reverse transcription into complementary DNA (cDNA). Quantitative real-time PCR (qRT-PCR) was performed using the SYBR Green fluorescence quantitative method. The relative expression levels of target genes were normalized to the internal reference gene GAPDH and calculated relative to the blank control group. All specific primer sequences used in this study are listed in Table S4.

### 4.9. Western Blotting

Cellular total proteins were extracted using RIPA lysis buffer. Protein samples were separated by 10% SDS-PAGE electrophoresis at 120 V and electro transferred onto polyvinylidene fluoride (PVDF) membranes (Millipore, Bedford, MA, USA). The membranes were blocked with 5% skim milk powder solution at room temperature for 1 h, then incubated overnight at 4 °C with the following primary antibodies (diluted in TBST): phospho-p65 (3033S, Cell Signaling Technology, Danvers, MA, USA; 1:1000), p65 (8242S, Cell Signaling Technology, Danvers, MA, USA; 1:2000), phospho-GSK3β (Tyr216) (ab75745, Abcam, Cambridge, UK; 1:2000), GSK3β (12456S, Cell Signaling Technology, Danvers, MA, USA; 1:2000), and GAPDH (2118S, Cell Signaling Technology, Danvers, MA, USA; 1:5000). After washing with TBST, the membranes were incubated with HRP-conjugated secondary antibody (RGAR001, Proteintech, Wuhan, China; 1:5000) for 1 h at room temperature. Protein bands were visualized using enhanced chemiluminescence (ECL) detection reagent (Pierce, Holmdel, NJ, USA). The band intensity was quantified using Image Lab software (Version 6.0, Bio-Rad Laboratories, Hercules, CA, USA) for statistical analysis.

### 4.10. Statistical Analysis

All statistical analyses were performed using SPSS Statistics 21.0. Data are expressed as mean ± standard deviation. Intergroup differences were assessed by one-way ANOVA, followed by post hoc comparisons using the least significant difference (LSD) test for groups with homogeneous variances and Dunnett’s T3 test for groups with heterogeneous variances (equality of variances evaluated by Levene’s test). A two-tailed *p* < 0.05 was considered statistically significant.

## Figures and Tables

**Table 1:** Digital DNA–DNA hybridization (dDDH) and average nucleotide identity (ANI) values of RerS4 and its most closely related *Streptomyces* species.

Species	RerS4	
	ANI	dDDH
*S. lavendulae* subsp. *grasserius* JCM 4056	85.87	31.9
*S. lavendulae* subsp. *colombiensis* NBRC 13454	85.30	31.6
*S. lavendulae* subsp. *lavendulae* NRRL B-2774^T^	85.46	31.7
*S. racemochromogenes* NRRL B-5430^T^	86.02	31.4
*S. polychromogenes* NBRC 13072^T^	84.62	30
*S. yangpuensis* fd2-tb^T^	83.96	28.7
*S. flavotricini* NRRL B-5419^T^	83.50	28.8
*S. amritsarensis* MTCC 11845^T^	83.80	28.9
*S. katrae* NRRL ISP-5550^T^	84.65	31.2
*S. globosus* LMG 19896^T^	83.00	26.8
*S. toxytricini* NBRC 12823^T^	82.87	26.7

**Table 2:** ^1^H and ^13^C NMR data of compound **1** in MeOD.

Position	δ_C_	δ_H_ (int., mult., *J* in Hz)
C1	179.6	-
C2	36.5	2.19 (t, *J* = 7.4 Hz, 2H)
C3	26.9	1.60 (t, *J* = 7.4 Hz, 2H)
C4–C9, C14–C18	30.3–30.8	1.34–1.29 (m, 22H)
C10, C13	28.1	2.03 (q, *J* = 6.9 Hz, 4H)
C11, C12	130.8	5.34 (dd, *J* = 10.0, 6.9 Hz, 2H)
C19	33.1	1.34–1.29 (m, 2H)
C20	23.7	1.34–1.29 (m, 2H)
C21	14.4	0.90 (t, *J* = 7.0 Hz, 3H)
C1′	160.5	-
C2′, C6′	116.4	6.76 (d, *J* = 8.7 Hz, 2H)
C3′, C5′	132.6	7.33 (d, *J* = 8.7 Hz, 2H)
C4′	136.7	-

## Data Availability

The original contributions presented in this study are included in the article and Supplementary Material. Further inquiries can be directed to the corresponding authors.

## Supplementary Materials

The following supporting information can be downloaded at https://www.mdpi.com/article/10.3390/molecules31213869/s1, Table S1: Differential morphological properties between strain RerS4 and most closely related species of the genus *Streptomyces*; Table S2: Differential phenotypic properties between strain RerS4 and most closely related species of the genus *Streptomyces*; Table S3: Fermentation medium used in this study; Table S4: Primers used in this study; Figure S1: HPLC of compound **1**; Figure S2: HRESIMS of compound **1**; Figure S3: ^1^H spectrum of compound **1**; Figure S4: ^13^C spectrum of compound **1**; Figure S5: HSQC spectrum of compound **1**; Figure S6: ^1^H-^1^H cosy spectrum of compound **1**; Figure S7: HMBC spectrum of compound **1**; Figure S8: SciFinder analysis of compound **1**; Figure S9: HPLC of compound **5**; Figure S10: HRESIMS of compound **5**; Figure S11: ^1^H spectrum of compound **5**; Figure S12: ^13^C spectrum of compound **5**; Figure S13: HSQC spectrum of compound **5**; Figure S14: ^1^H-^1^H cosy spectrum of compound **5**; Figure S15: HMBC spectrum of compound **5**; Figure S16: CCK8 assay of compound **1–5**; Figure S17: The deposit certificate of strain RerS4.
