# Supplementary material for: Streptomyces antiinflammaticus from Rehmannia glutinosa Produces Dual Immunomodulatory Metabolites That Upregulate IL-10 and Inhibit GSK3β/NF-κB
Source: Molecules. 2026 Oct 27;31(21):3869. doi: 10.3390/molecules31213869 (PMC13765442; doi:10.3390/molecules31213869)
Supplement: Supplementary file 1 [file molecules-31-03869-s001.zip › Supplementary fileS1.pdf]

Streptomyces antiinflammaticus from *Rehmannia glutinosa* produces dual immunomodulatory metabolites that upregulate IL-10 and inhibit GSK3β/NF-κB

Tong Wang[a], Yu Liang[a], Feiyan Yang[a], Yueqing Du[b], Jiaqi Li[a], Pengqiang Du[b], Yanqin Qin[a*], Hairong He[a*]

a, School of Pharmacy, Henan University of Chinese Medicine, Zhengzhou 450046, China

b, College of Plant Protection, Henan Agricultural University, Zhengzhou 450046, China

Corresponding Authors: Yanqin Qin and Hairong He (hhirong@163.com)

Running title: IL-10 and anti-inflammatory agents from *S. antiinflammaticus.*

Supplementary Materials

Table S1 Differential morphological properties between strain RerS4 and most closely related species of the genus *Streptomyces*.

Medium and characteristics	1	2	3	4	5	6	7	8	9
**ISP 2**									
Aerial mycelium color	sandy	sandy	sandy	sandy	pale brown	grey	pale brown	pale brown	light brown
Substrate mycelium color	golden yellow	golden yellow	golden yellow	golden yellow	golden yellow	brown	brown orange	golden yellow	brown
pigments	N	Y	Y	Y	Y	Y	Y	Y	Y
growth	+++	+++	+++	+++	+++	+++	+++	+++	+++
**ISP 3**									
Aerial mycelium color	white	sandy	pale brown	pale brown	light brown	sandy	pale brown	sandy	pale brown
Substrate mycelium color	beige	cream	beige	beige	cream	beige	light brown	pale brown	yellow
pigment	N	N	N	N	N	N	N	N	N
growth	+++	+++	+++	+++	+++	+++	+++	+++	+++
**ISP 4**									
Aerial mycelium color	white	pale brown	none	sandy	white	white	pale brown	sandy	cream
Substrate mycelium color	beige	beige	pale yellow	cream	cream	beige	sandy	beige	cream
pigment	N	N	N	N	N	N	N	N	N
growth	++	+++	+	+++	+++	++	++	+++	++
**ISP 5**									
Aerial mycelium color	none	none	none	none	none	none	none	none	none
Substrate mycelium color	white yellow	pale yellow	pale yellow	pale yellow	beige	beige	beige	beige	pale yellow
pigment	N	N	N	N	N	N	N	N	N
growth	+	+	+	+	+	+	+	+	+
**ISP 6**									
Aerial mycelium color	none	none	none	none	none	none	none	none	none
Substrate mycelium color	pale yellow	pale yellow	cream	pale yellow	pale yellow	cream	beige	cream	cream
pigment	N	N	Y	Y	Y	N	Y	N	N
growth	++	+	+	+	+	+	+	+	+
**ISP 7**									

**Medium and characteristics**	**1**	**2**	**3**	**4**	**5**	**6**	**7**	**8**	**9**
Aerial mycelium color	white	light brown	none	white	pale brown	sandy	pale brown	white	cream
Substrate mycelium color	dark brown	beige	beige	cream	brown orange	cream	cream	cream	brown orange
pigment	Y	N	N	N	N	++	N	N	Y
growth	++	++	++	++	+++	++	++	+++	+++
**nutrient agar**									
Aerial mycelium color	none	none	none	white	none	white	white	white	white
Substrate mycelium color	pale yellow	pale yellow	pale yellow	beige	pale yellow	beige	beige	beige	cream
pigment	N	N	N	N	N	N	N	N	++
growth	++	+	++	++	+	++	++	++	++
**Bennet**									
Aerial mycelium color	white pink	none	none	none	none	none	white	none	none
Substrate mycelium color	pink	beige	beige	beige	cream	beige	pale brown	beige	cream
pigment	N	N	N	N	N	N	N	N	N
growth	++	++	++	++	+	++	+++	++	++

+ poor growth; ++ moderate growth; +++ abundant growth; N, no; Y, yes

Strains: 1, RerS4; 2, *S. yangpuensis* fd2-tb^{T}; 3, *S. amritsarensis* JCM19660^{T}; 4, *S. flavotricini* NBRC 12770^{T}; 5, *S. toxytricini* NBRC 12823^{T}; 6, *S. racemochromogenes* NRRL B-5430^{T}; 7, *S. katrae* NBRC 13447^{T}; 8, *S. polychromogenes* NBRC 13072^{T}; 9, *S. globosus* LMG 19896^{T}

Data for strain RerS4 are form this study, and data for strains 2-9 from Tang *et al*.

Table S2 Differential phenotypic properties between strain RerS4 and most closely related species of the genus *Streptomyces*.

**Characteristic**	**1**	**2**	**3**	**4**	**5**	**6**	**7**	**8**	**9**
Growth on sole carbon sources (1 %, w/v)									
L-Arabinose	-	-	+	-	-	-	-	-	-
D-Fructose	-	-	+	-	-	-	-	-	-
D-Galactose	+	-	-	w	-	-	-	-	-
D-Glucose	+	+	+	+	+	-	-	+	+
Maltose	+	+	+	+	+	-	-	+	+
L-Rhamnose	+	+	-	+	+	-	-	-	-
Sucrose	+	-	-	-	-	+	+	+	-
Growth on sole nitrogen sources (0.1 %, w/v)									
L-Serine	-	-	-	-	+	-	+	+	+

Characteristic	1	2	3	4	5	6	7	8	9
L-Tyrosine	-	-	-	-	-	-	+	+	-
Alanine	+	-	-	-	+	-	+	+	-
Arginine	+	+	+	+	+	+	+	+	-
Leucine	+	-	-	-	-	+	+	+	-
Isoluecine	+	-	-	-	-	-	+	+	-
Glycine	-	w	-	+	+	+	+	+	+
L-Histidine	+	-	-	-	-	-	+	-	-
L-Proline	-	-	-	-	+	+	+	+	-
L-Threonine	-	-	-	-	+	-	+	+	-
Growth at:									
10 °C	w	w	w	+	w	w	-	+	-
37 °C	+	+	+	+	w	w	+	+	+
40 °C	+	+	w	+	w	w	+	+	w
pH 5.0	+	+	+	+	+	+	+	+	w
pH 6.0	+	+	+	+	+	+	+	+	w
pH 8.0	+	+	w	+	+	+	+	+	+
pH 9.0	+	+	w	+	+	+	+	+	+
pH 10.0	+	+	w	w	w	w	w	+	+
Growth with NaCl (%, w/v)									
1	+	+	w	+	w	+	w	+	w
2	+	+	w	w	w	w	w	+	w
4	-	w	w	w	w	w	-	w	w
5	-	-	-	-	-	-	-	-	w

-, negative; +, positive; W, weak growth.

Strains: 1, RerS4; 2, *S. yangpuensis* fd2-tb^T; 3, *S. amritsarensis* JCM19660^T; 4, *S. flavotricini* NBRC 12770^T; 5, *S. toxytricini* NBRC 12823^T; 6, *S. racemochromogenes* NRRL B-5430^T; 7, *S. katrae* NBRC 13447^T; 8, *S. polychromogenes* NBRC 13072^T; 9, *S. globosus* LMG 19896^T

Data for strain RerS4 are form this study, and data for strains 2-9 from Tang *et al*.

Table S3 Fermentation medium used in this study.

Medium	Ingredients and content
GYM I	skim milk powder 0.1%, yellow bean cake flour 2%, sucrose 8%, $K_2HPO_4·3H_2O$ 0.1%, $FeSO_4·7H_2O$ 0.1%, $CaCO_3$ 0.3%, pH 7.2-7.4
GYM II	D-Glucose, 0.4%; Malt extract, 1%; Yeast extract, 0.4%; Soluble starch, 4%; $CaCO_3$, 0.2%; Trace element solution, 1 mL/L ($FeSO_4·7H_2O$ 0.1%, $MnCl_2·4H_2O$ 0.1%, $ZnSO_4·7H_2O$ 0.1%). The pH 7.2–7.4.
ISP 2	yeast extract powder 0.4%, malt extract 1%, D-glucose 0.4%, pH7.2-7.4
HZ-2	yeast extract powder 0.4%, $FeSO_4·7H_2O$ 0.1%, malt extract 0.2%, D- glucose 1%, soluble starch 4%, $CaCO_3$ 0.2%, $MnCl_2·4H_2O$ 0.1%, pH 7.2-7.4
MD	yeast extract powder 1%, D-glucose 1%, $K_2HPO_4·3H_2O$ 0.05%, $MgSO_4·7H_2O$ 0.05%, pH 7.2

HDBF	yeast extract powder 0.4%, maltodextrin 1%, D-glucose 1%, soluble starch 4%, trace element solution 0.1mL/L ($FeSO_4 \cdot 7H_2O$ 0.1%, $MnCl_2 \cdot 4H_2O$ 0.1%, $ZnSO_4 \cdot 7H_2O$ 0.1%), pH 7.2-7.4
MB	D-glucose 1%, soluble starch 0.5%, yeast extract powder 0.2%, NaCl 0.4%, peptone 0.2%, $K_2HPO_4 \cdot 3H_2O$ 0.05%, $MgSO_4 \cdot$7H2O 0.05%, $CaCO_3$ 0.2%, pH 7.2
H9	soluble starch 2%, cottonseed meal powder 1%, yeast extract powder 0.5%, maltodextrin 2%, malt extract 0.5%, $MgSO_4 \cdot 7H_2O$ 0.2%, NaCl 0.2 %, $CaCO_3$ 0.2%, pH 7.0-7.2

Table S4 Primers used in this study.

Primers	Sequences
IL-6-F	5'- AGACAGCCACTCACCTCTTCAG -3'
IL-6-R	5'- TTCTGCCAGTGCCCTCTTTG -3'
IL-1β-F	5'- ATGATGGCTTATTACAGTGGCAA -3'
IL-1β-R	5'- GTCGGAGATTCGTAGCTGGA -3'
TNF-α-F	5'- CCTCTCTCTAATCAGCCCTCTG -3'
TNF-α-R	5'- GAGGACCTGGGAGTAGATGAG -3'
IL-10-F	5'- TGAGAACCAAGACCCAGACATCA -3'
IL-10-R	5'- GCCTTGCTCTTGTTTTCACAGG -3'
GAPDH-F	5'- GAAGGTGAAGGTCGGAGTC -3'
GAPDH-R	5'- GAAGATGGTGATGGGATTTC -3'

Figure legends

Figure S1 HPLC of compound **1.**

Figure S2 HRESIMS of compound **1**.

Figure S3 ^{1}H spectrum of compound **1**.

Figure S4 ^{13}C spectrum of compound **1**.

Figure S5 HSQC spectrum of compound **1**.

Figure S6 ^{1}H-^{1}H cosy spectrum of compound **1**.

Figure S7 HMBC spectrum of compound **1**.

Figure S8 SciFinder analysis of compound **1**.

Figure S9 HPLC of compound **5.**

Figure S10 HRESIMS of compound **5**.

Figure S11 ^{1}H spectrum of compound **5**.

Figure S12 ^{13}C spectrum of compound **5**.

Figure S13 HSQC spectrum of compound **5**.

Figure S14 ^{1}H-^{1}H cosy spectrum of compound **5**.

Figure S15 HMBC spectrum of compound **5**.

Figure S16 CCK8 assay of compound **1-5**.

Figure S17 The deposit certificate of strain RerS4.

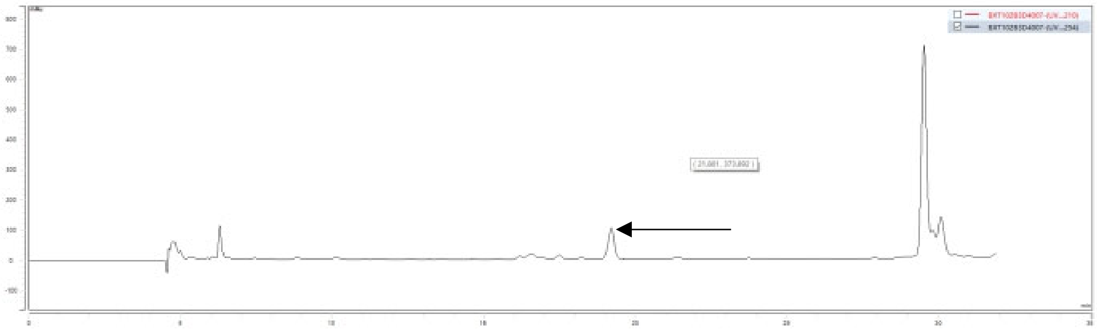

Figure S1 HPLC of compound **1.**

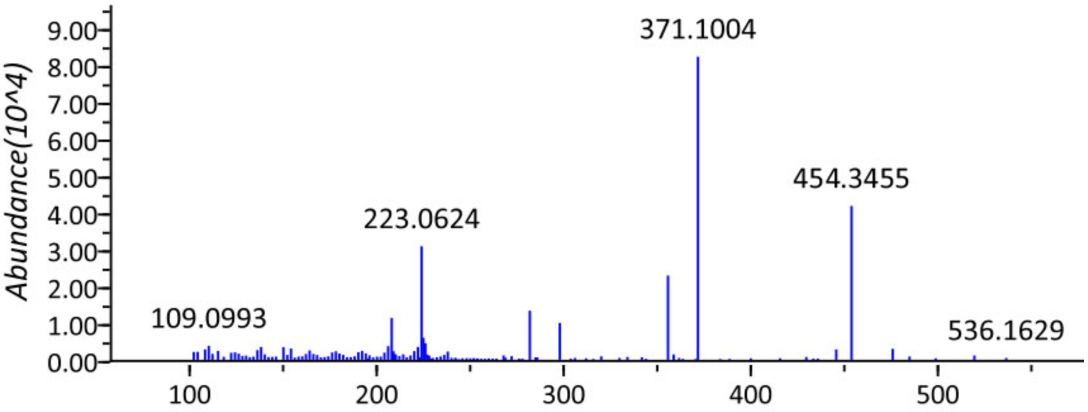

Figure S2 HRESIMS of compound **1**.

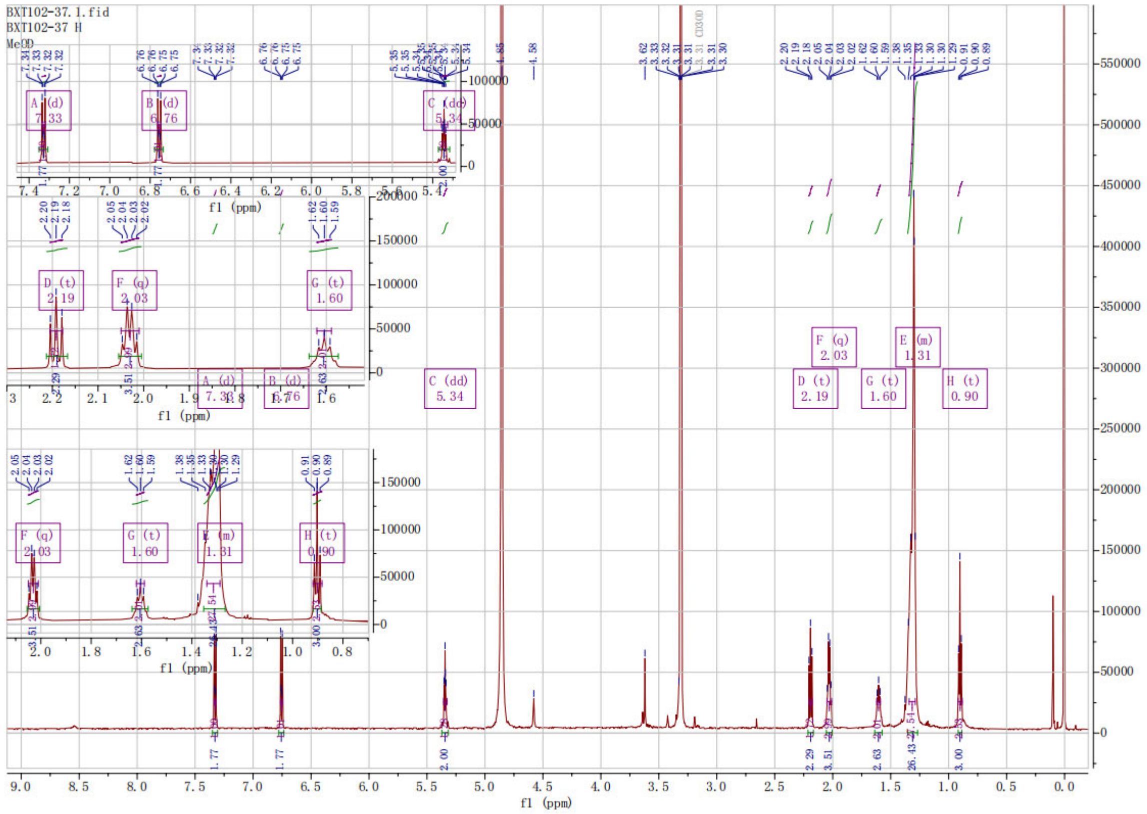

Figure S3 ^{1}H spectrum of compound **1**.

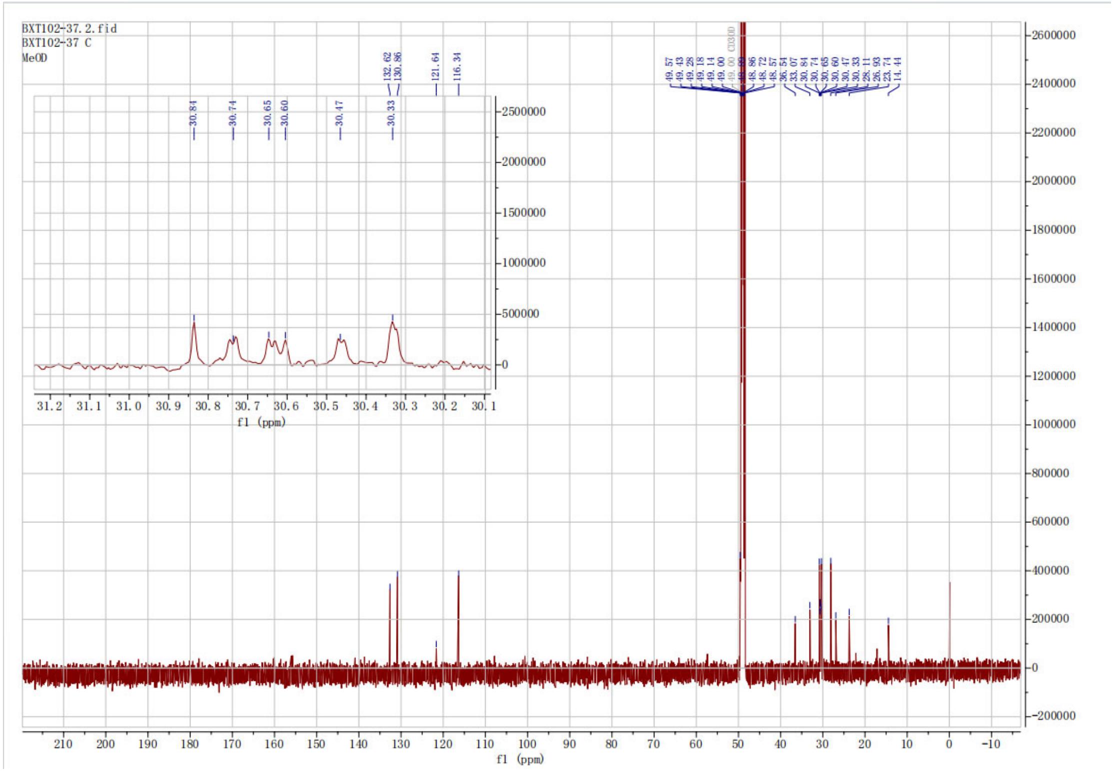

Figure S4 ^{13}C spectrum of compound **1**.

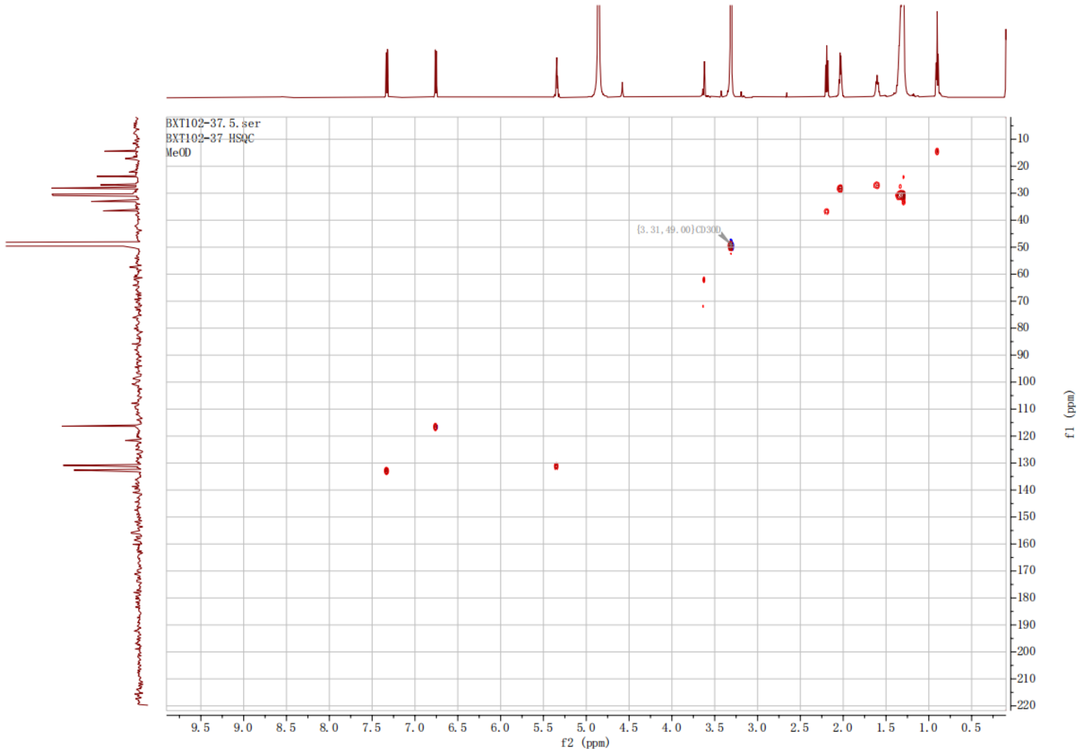

Figure S5 HSQC spectrum of compound **1**.

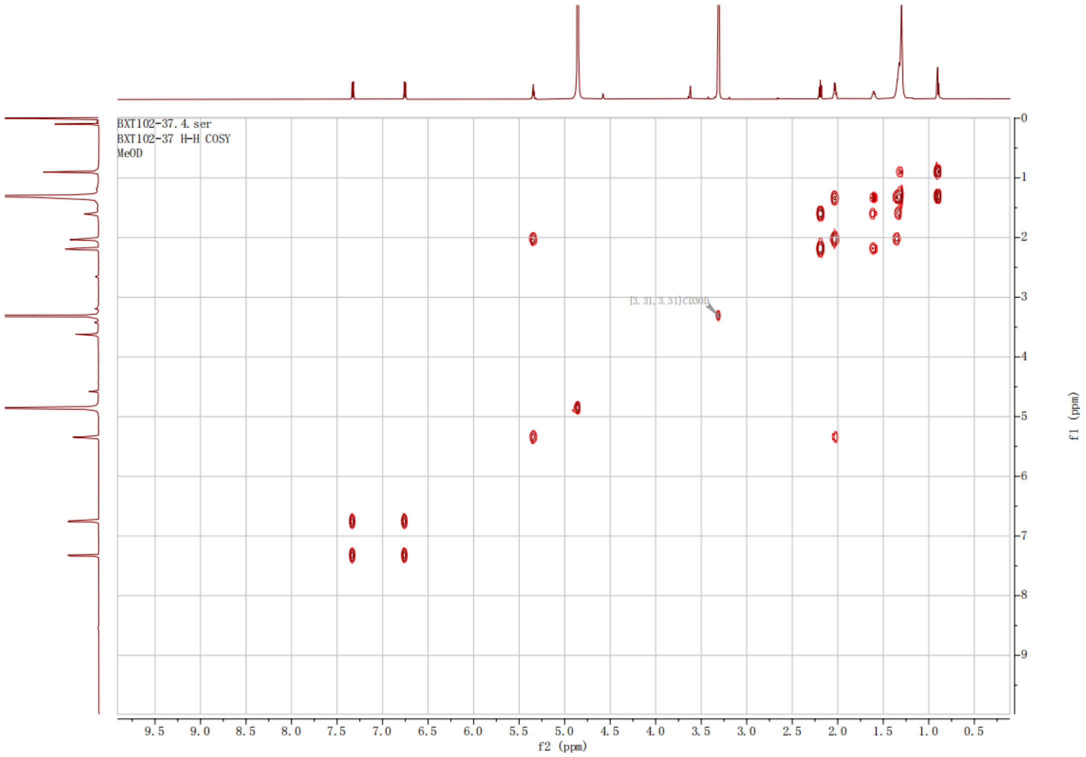

Figure S6 ^{1}H-^{1}H cosy spectrum of compound **1**.

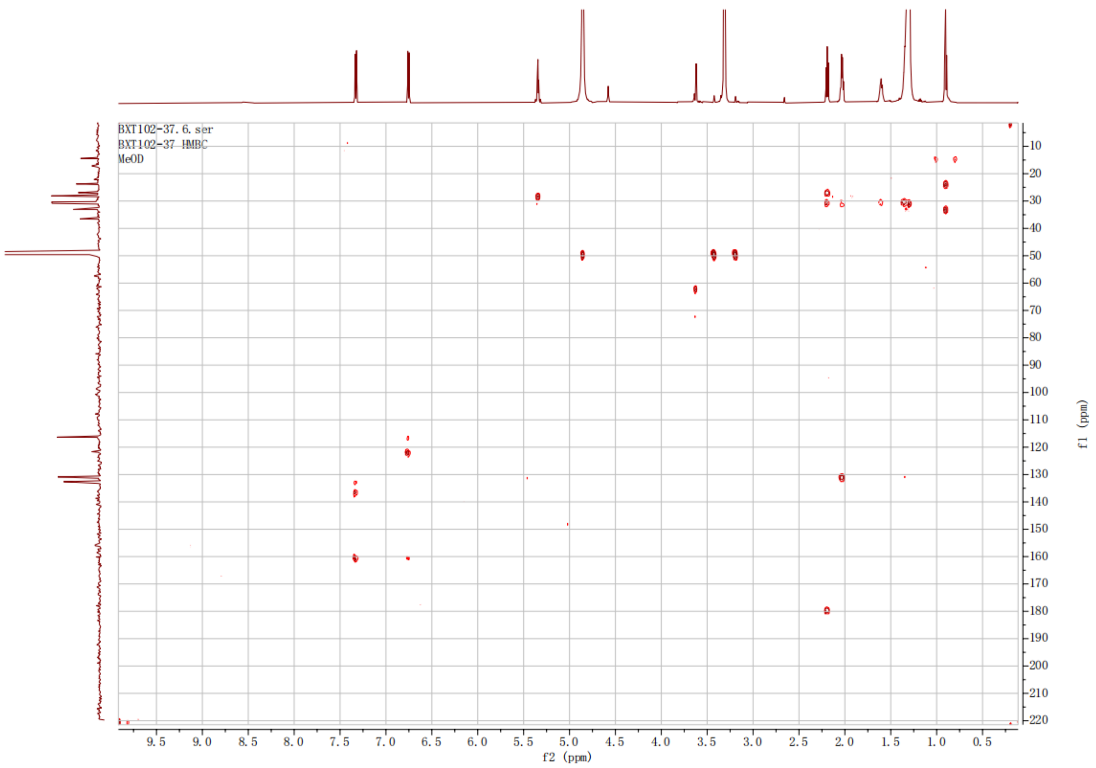

Figure S7 HMBC spectrum of compound **1**.

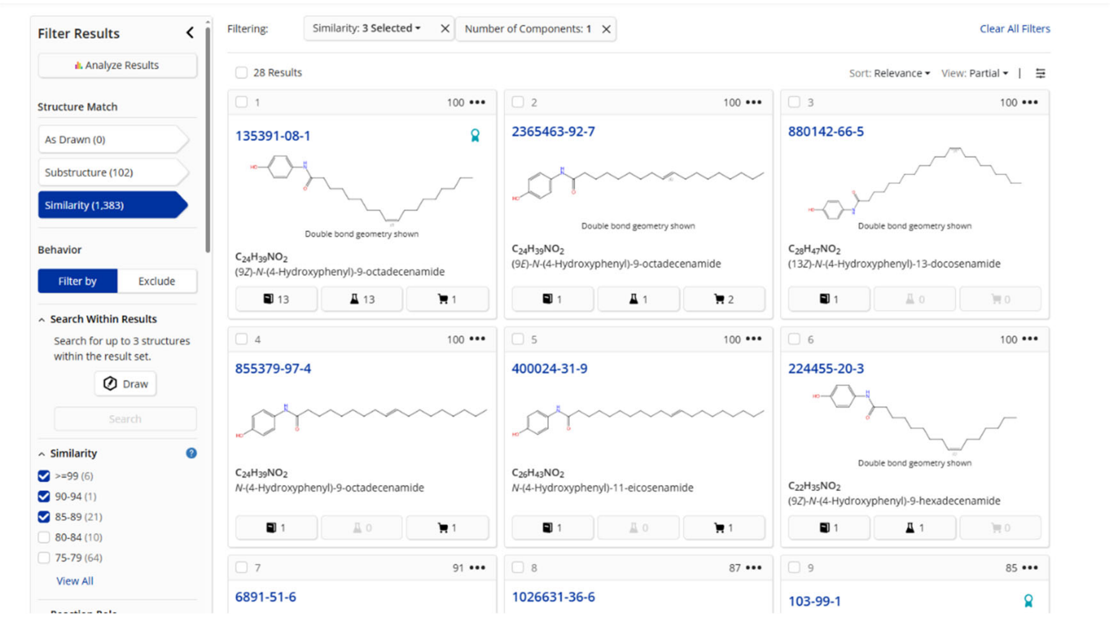

Figure S8 SciFinder analysis of compound **1**.

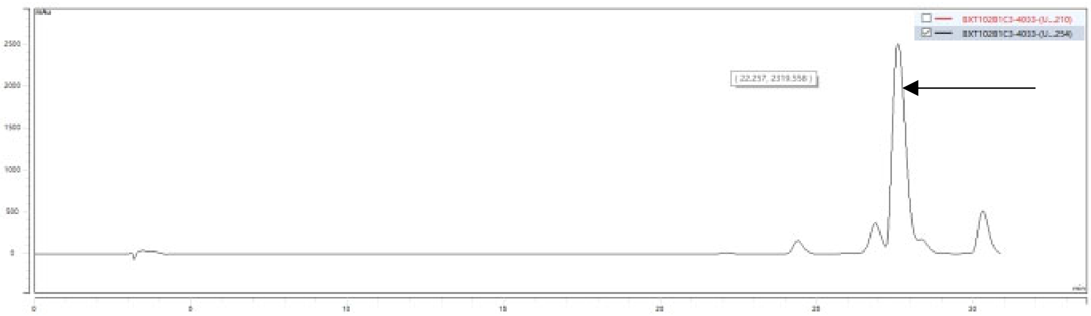

Figure S9 HPLC of compound **5.**

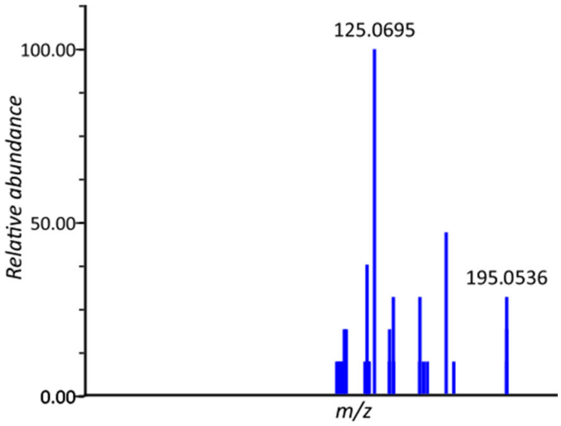

Figure S10 HRESIMS of compound **5**.

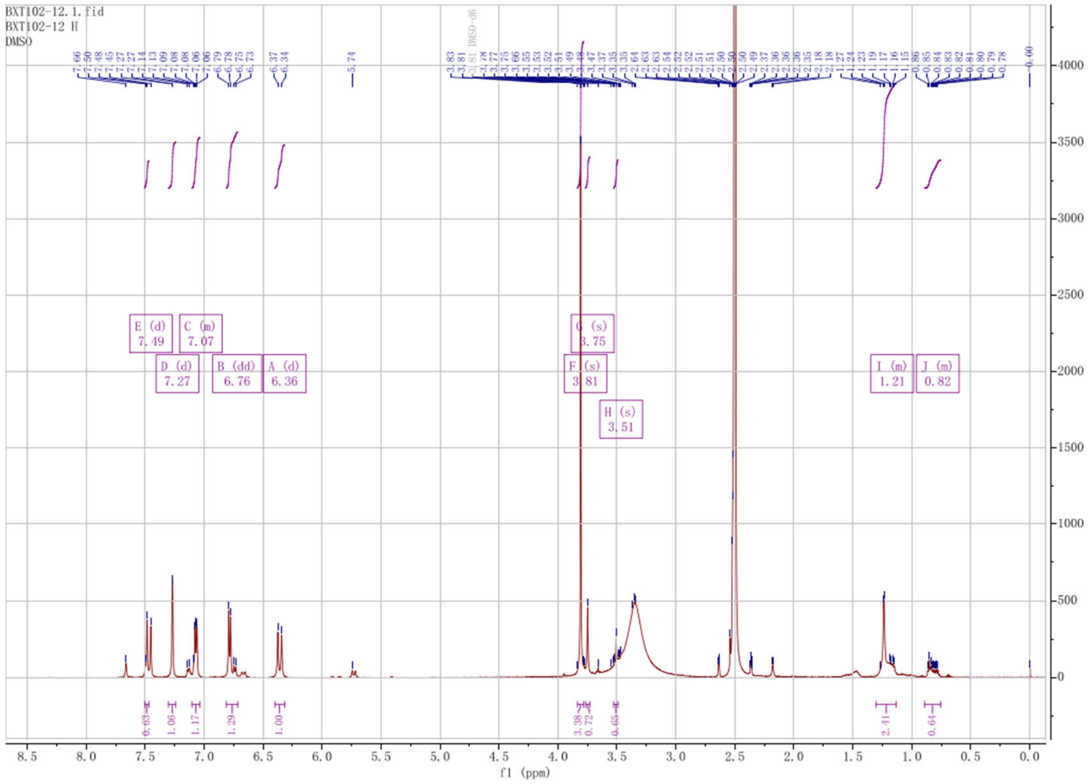

Figure S11 ^{1}H spectrum of compound **5**.

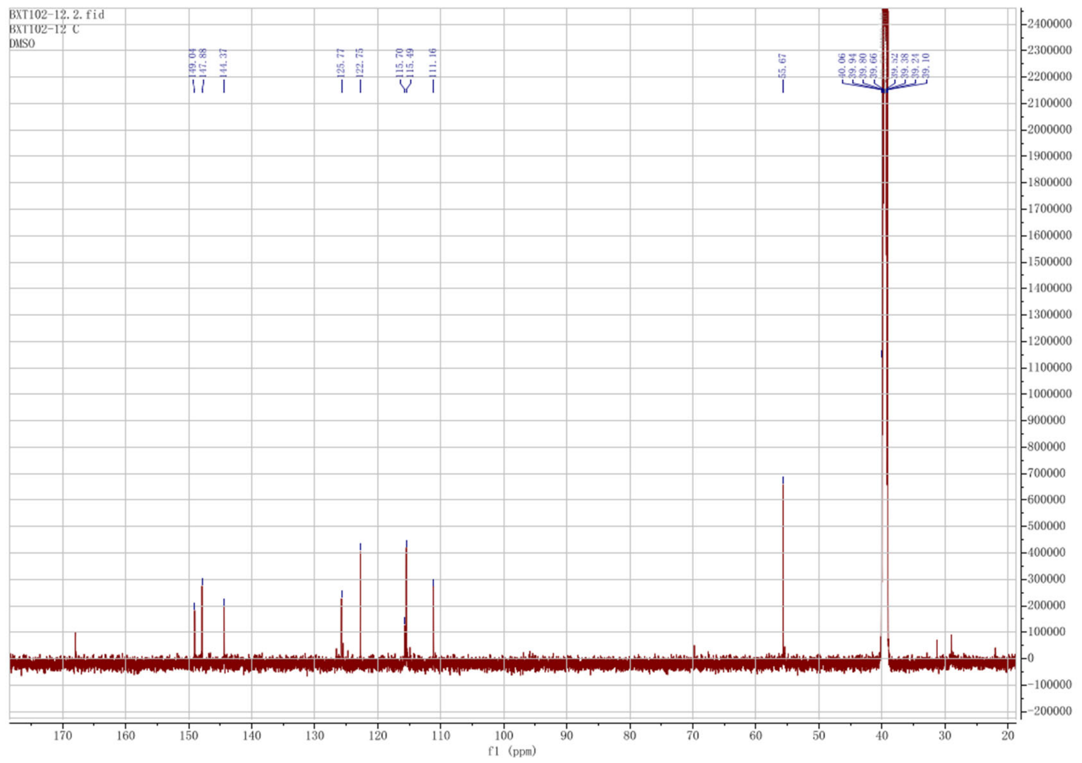

Figure S12 ^{13}C spectrum of compound **5**.

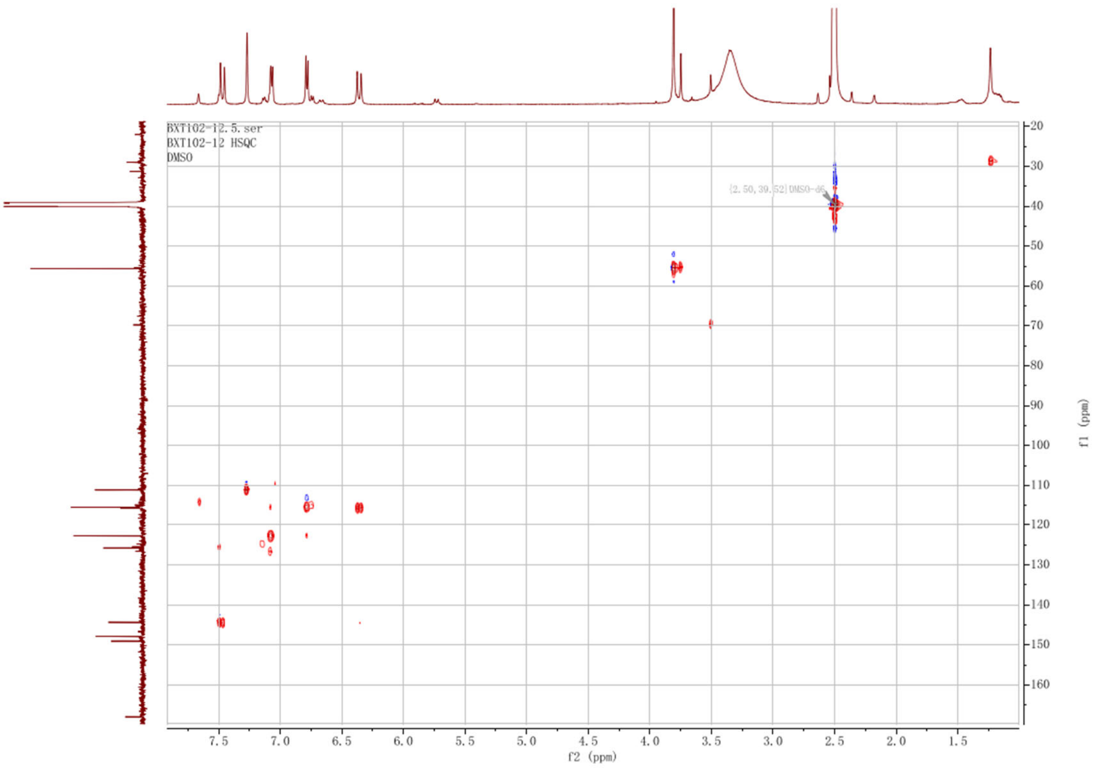

Figure S13 HSQC spectrum of compound **5**.

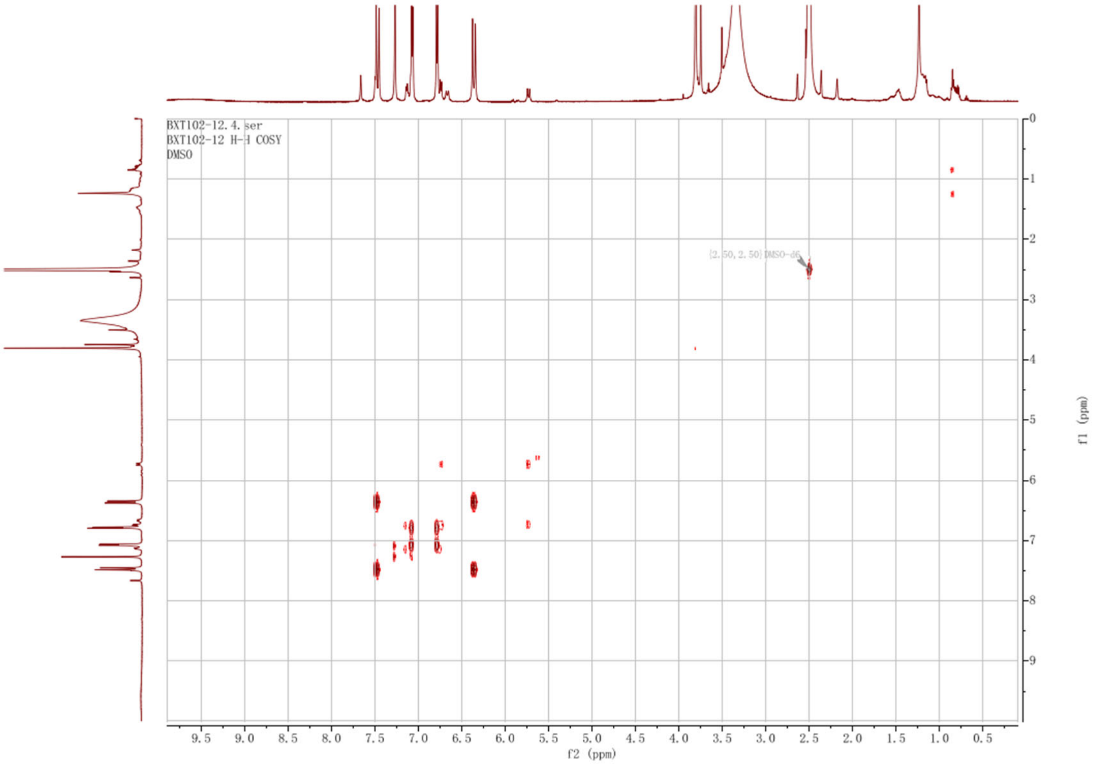

Figure S14 ^{1}H-^{1}H cosy spectrum of compound **5**.

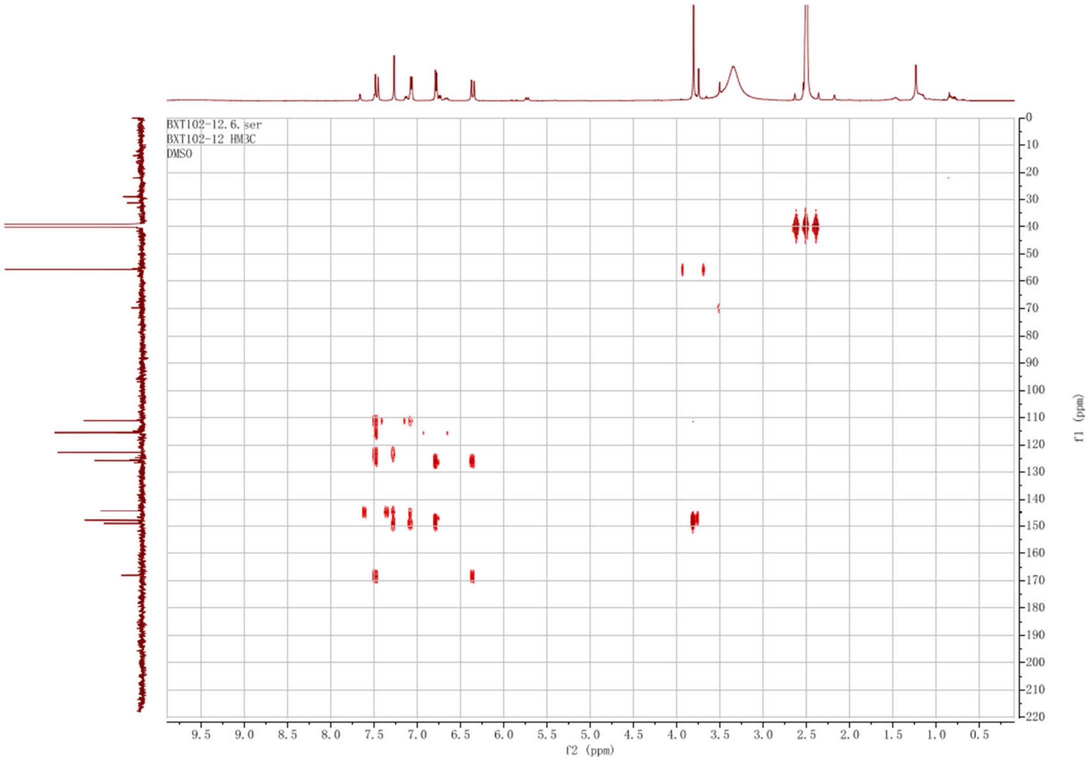

Figure S15 HMBC spectrum of compound **5**.

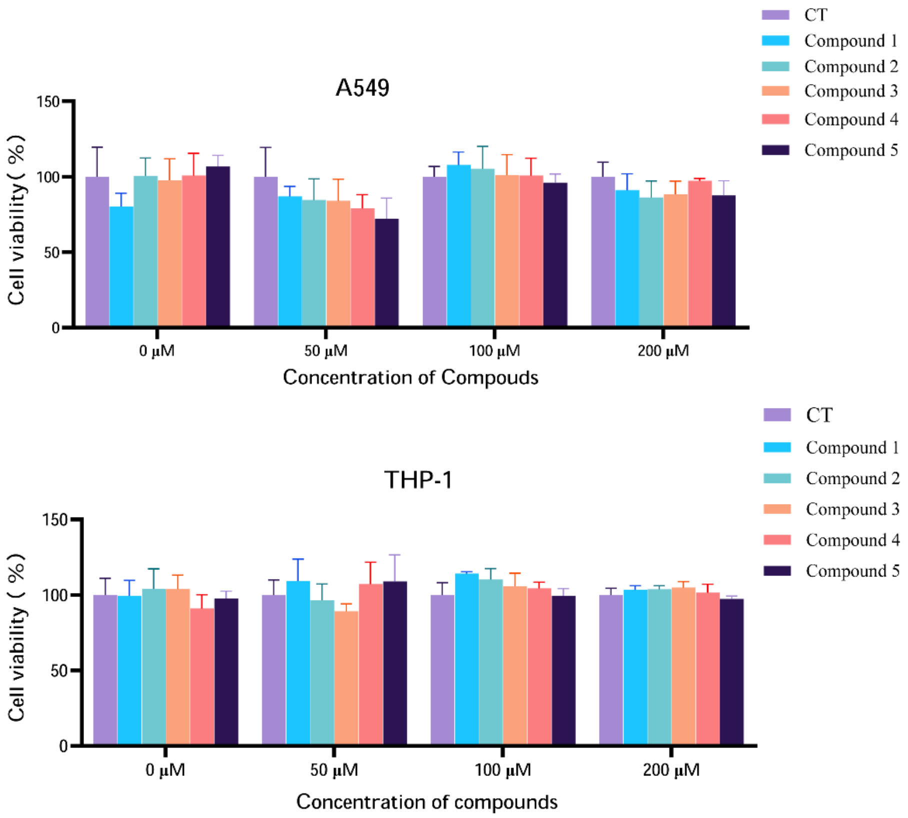

Figure S16 CCK8 assay of compound **1-5**.

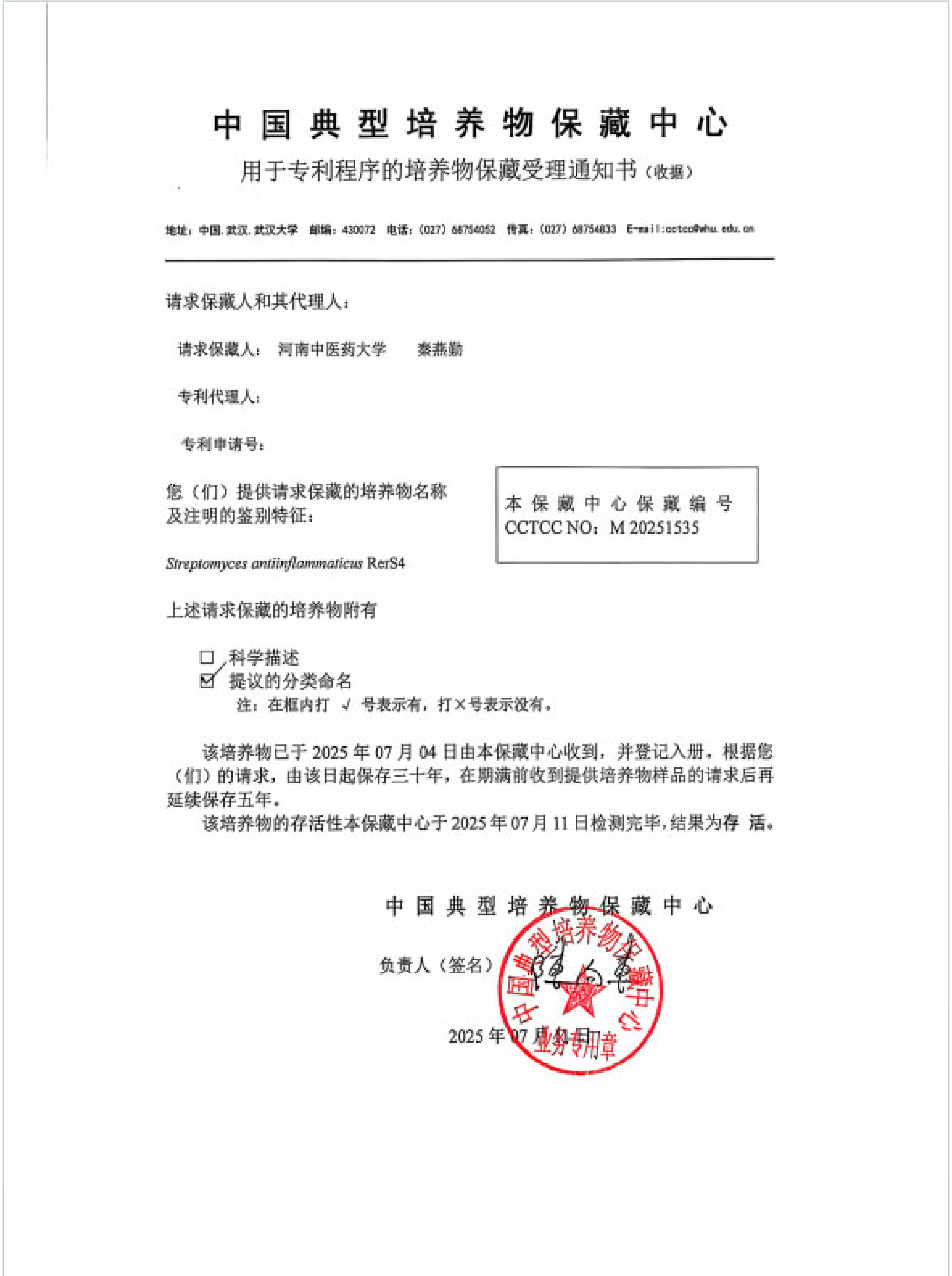

中 国 典 型 培 养 物 保 藏 中 心

用于专利程序的培养物保藏受理通知书（收据）

地址：中国.武汉.武汉大学　邮编：430072　电话：（027）68754052　传真：（027）68754833　E-mail:cctcc@whu.edu.cn

请求保藏人和其代理人：

请求保藏人：　河南中医药大学　　秦燕勤

专利代理人：

专利申请号：

您（们）提供请求保藏的培养物名称及注明的鉴别特征：

Streptomyces antiinflammaticus RerS4

本保藏中心保藏编号
CCTCC NO：M 20251535

上述请求保藏的培养物附有

☐ 科学描述
☑ 提议的分类命名

注：在框内打 √ 号表示有，打×号表示没有。

该培养物已于 2025 年 07 月 04 日由本保藏中心收到，并登记入册。根据您（们）的请求，由该日起保存三十年，在期满前收到提供培养物样品的请求后再延续保存五年。

该培养物的存活性本保藏中心于 2025 年 07 月 11 日检测完毕，结果为存 活。

中 国 典 型 培 养 物 保 藏 中 心

负责人（签名）

2025 年 07 月 [illegible] 日

Figure S17 The deposit certificate of strain RerS4. This document is an official Chinese receipt issued by the China Center for Type Culture Collection (CCTCC). The key information on the certificate is translated as follows:

Depository institution: China Center for Type Culture Collection (CCTCC, Wuhan University, Wuhan, China)

CCTCC NO: M 20251535

Depositor: Yanqin Qin, Henan University of Chinese Medicine

Strain: Streptomyces antiinflammaticus RerS4

Supplied documents: Proposed taxonomic designation
Receipt date of the strain: July 04, 2025
Preservation term: 30 years from the receipt date; an additional 5-year extension is available upon request before expiry
Viability test date: July 11, 2025
Viability test result: viable
