# Supplementary material for: Streptomyces antiinflammaticus from Rehmannia glutinosa Produces Dual Immunomodulatory Metabolites That Upregulate IL-10 and Inhibit GSK3β/NF-κB
Source: Molecules. 2026 Oct 27;31(21):3869. doi: 10.3390/molecules31213869 (PMC13765442; doi:10.3390/molecules31213869)
Supplement: Supplementary file 1 [file molecules-31-03869-s001.zip › supplementary fileS2.pdf]

**Supplementary file S2**

**Because there are many original films, we put them in tables to show clearly. All Western blot experiments were performed in triplicate; the left panel shows two replicates, and the right panel shows one replicate or two replicates.**

**Original films of western blot shown in Figure 8.**

| | |
|---|---|
| Original films of p-P65 (65KDa) of A549 cells. | |
| Bands: Marker, Marker, CT, TNF-α, 200 μM, 150 μM, 100 μM, Marker, CT, TNF-α, 200 μM, 150 μM, 100 μM, Marker | Bands: Marker, CT, TNF-α, 200 μM, 150 μM, 100 μM, Marker |
| 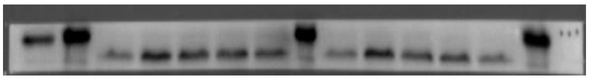 | 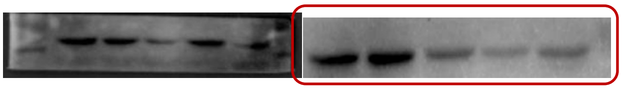 |
| Original films of P65 (65KDa) of A549 cells. | |
| Bands: Marker, Marker, CT, TNF-α, 200 μM, 150 μM, 100 μM, Marker, CT, TNF-α, 200 μM, 150 μM, 100 μM, Marker | Bands: Marker, CT, TNF-α, 200 μM, 150 μM, 100 μM |
| 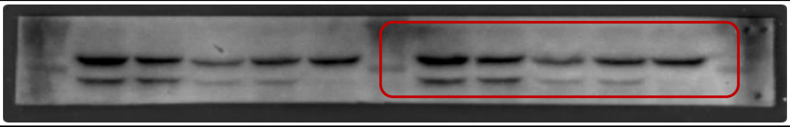 | 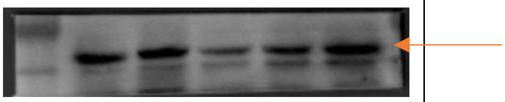 |
| Original films of p-GSK3β (46KDa) of A549 cells. | |
| Bands: Marker, Marker, CT, TNF-α, 200 μM, 150 μM, 100 μM, Marker, CT, TNF-α, 200 μM, 150 μM, 100 μM, Marker | Bands: Marker, Marker, CT, TNF-α, 200 μM, 150 μM, 100 μM, Marker, CT, TNF-α, 200 μM, 150 μM, 100 μM, Marker |
| 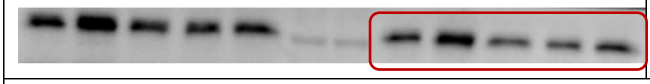 | 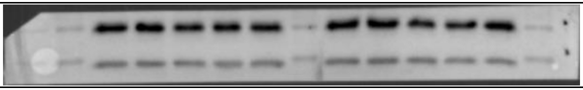 |
| Original films of GSK3β (46KDa) of A549 cells. | |
| Bands: Marker, Marker, CT, TNF-α, 200 μM, 150 μM, 100 μM, Marker, CT, TNF-α, 200 μM, 150 μM, 100 μM, Marker | Bands: Marker, CT, TNF-α, 200 μM, 150 μM, 100 μM, Marker |
| 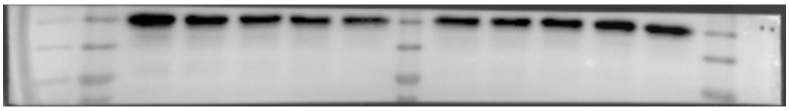 | 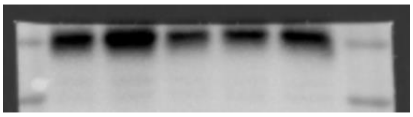 |
| 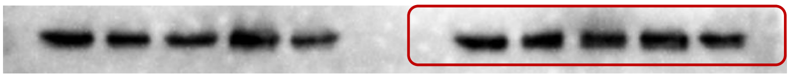 | |
| Original films of GAPDH (36KDa) of A549 cells. | |
| Bands: Marker, Marker, CT, TNF-α, 200 μM, 150 μM, 100 μM, Marker, CT, TNF-α, 200 μM, 150 μM, 100 μM, Marker | Bands: Marker, CT, TNF-α, 200 μM, 150 μM, 100 μM |
| 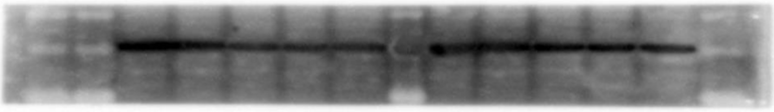 | 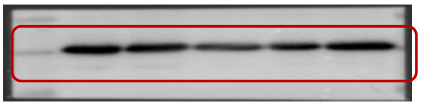 |
| Original films of p-P65 (65KDa) of THP-1 cells. | |
| Bands: Marker, Marker, CT, TNF-α, 200 μM, 150 μM, 100 μM, Marker, CT, TNF-α, 200 μM, 150 μM, 100 μM, Marker | Bands: Marker, CT, TNF-α, 200 μM, 150 μM, 100 μM, Marker |

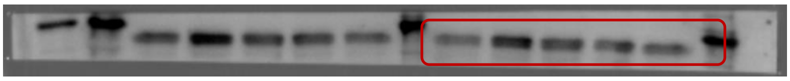
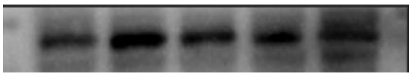

Original films of P65 (65KDa) of THP-1 cells.

Bands: Marker, Marker, CT, TNF-α, 200 μM, 150 μM, 100 μM, Marker, CT, TNF-α, 200 μM, 150 μM, 100 μM, Marker

Bands: CT, TNF-α, 200 μM, 150 μM, 100 μM, Marker, CT, TNF-α, 200 μM, 150 μM, 100 μM,

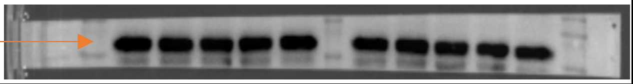
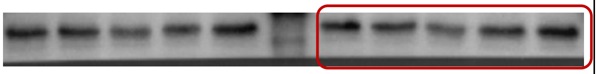

Original films of p-GSK3β (46KDa) of THP-1 cells.

Bands: Marker, Marker, CT, TNF-α, 200 μM, 150 μM, 100 μM, Marker, CT, TNF-α, 200 μM, 150 μM, 100 μM

Bands: Marker, CT, TNF-α, 200 μM, 150 μM, 100 μM, Marker

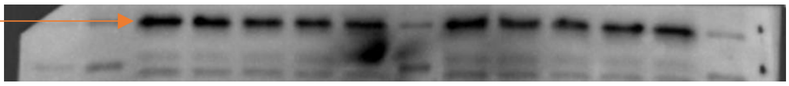
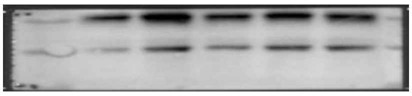
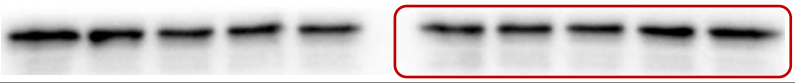

Original films of GSK3β (46KDa) of THP-1 cells.

Bands: Marker, Marker, CT, TNF-α, 200 μM, 150 μM, 100 μM, Marker, CT, TNF-α, 200 μM, 150 μM, 100 μM, Marker

Bands: Marker, Marker, CT, TNF-α, 200 μM, 150 μM, 100 μM, Marker, CT, TNF-α, 200 μM, 150 μM, 100 μM, Marker

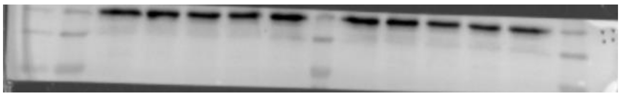
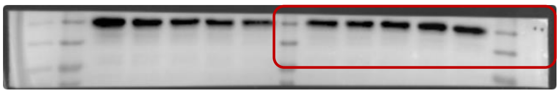

Original films of GAPDH (36KDa) of THP-1 cells.

Bands: Marker, Marker, CT, TNF-α, 200 μM, 150 μM, 100 μM, Marker, CT, TNF-α, 200 μM, 150 μM, 100 μM, Marker

Bands: Marker, Marker, CT, TNF-α, 200 μM, 150 μM, 100 μM, Marker

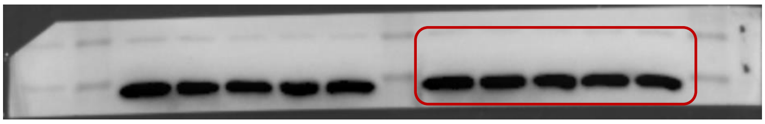
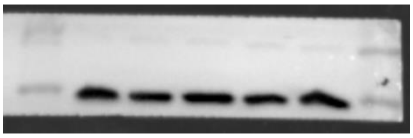

## Original films of western blot shown in Figure 9.

Original films of p-P65 (65KDa) of A549 cells.

Bands: Marker, CT, TNF-α, 200 μM, 150 μM, 100 μM, Marker, CT, TNF-α, 200 μM, 150 μM, 100 μM, Marker

Bands: Marker, CT, TNF-α, 200 μM, 150 μM, 100 μM Marker, CT, TNF-α, 200 μM, 150 μM, 100 μM

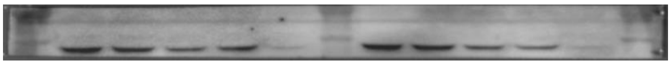
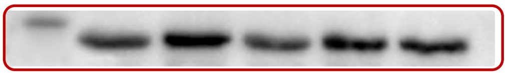

Original films of P65 (65KDa) of A549 cells.

Bands: Marker, Marker, CT, TNF-α, 200 μM, 150 μM, 100 μM, Marker, CT, TNF-α, 200 μM, 150 μM, 100 μM, Marker

Bands: Marker, CT, TNF-α, 200 μM, 150 μM, 100 μM Marker, CT, TNF-α, 200 μM, 150 μM, 100 μM

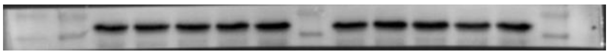
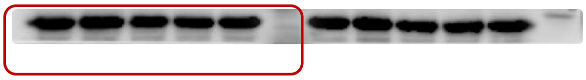

<table>
<tr><td colspan="2">Original films of p-GSK3β (46KDa) of A549 cells.</td></tr>
<tr><td>Bands: Marker, Marker, CT, TNF-α, 200 μM, 150 μM, 100 μM, Marker, CT, TNF-α, 200 μM, 150 μM, 100 μM, Marker</td><td>Bands: Marker, CT, TNF-α, 200 μM, 150 μM, 100 μM, Marker</td></tr>
<tr><td>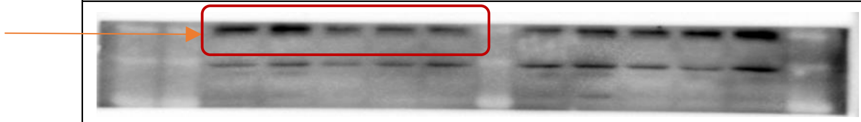</td><td>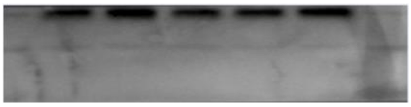</td></tr>
<tr><td colspan="2">Original films of GSK3β (46KDa) of A549 cells.</td></tr>
<tr><td>Bands: Marker, CT, TNF-α, 200 μM, 150 μM, 100 μM, Marker, CT, TNF-α, 200 μM, 150 μM, 100 μM, Marker</td><td>Bands: Marker, CT, TNF-α, 200 μM, 150 μM, 100 μM, Marker</td></tr>
<tr><td>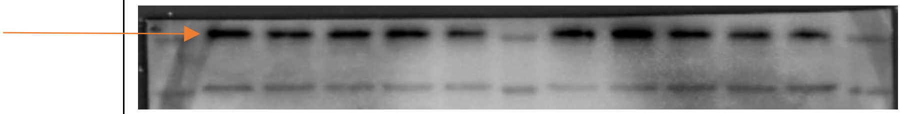</td><td>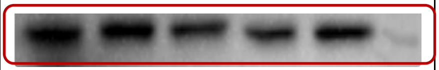</td></tr>
<tr><td colspan="2">Original films of GAPDH (36KDa) of A549 cells.</td></tr>
<tr><td>Bands: Marker, Marker, CT, TNF-α, 200 μM, 150 μM, 100 μM, Marker, CT, TNF-α, 200 μM, 150 μM, 100 μM, Marker</td><td>Bands: Marker, CT, TNF-α, 200 μM, 150 μM, 100 μM, Marker</td></tr>
<tr><td>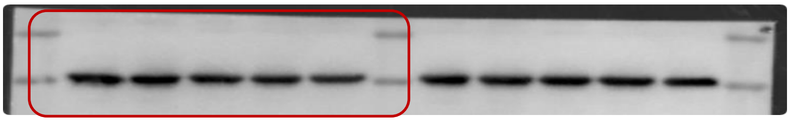</td><td>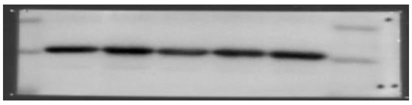</td></tr>
<tr><td colspan="2">Original films of p-P65 (65KDa) of THP-1 cells.</td></tr>
<tr><td>Bands: Marker, Marker, CT, TNF-α, 200 μM, 150 μM, 100 μM, Marker, CT, TNF-α, 200 μM, 150 μM, 100 μM, Marker</td><td>Bands: Marker, Marker, CT, TNF-α, 200 μM, 150 μM, 100 μM, Marker, CT, TNF-α, 200 μM, 150 μM, 100 μM, Marker</td></tr>
<tr><td>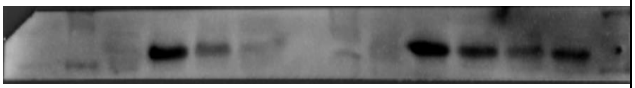</td><td>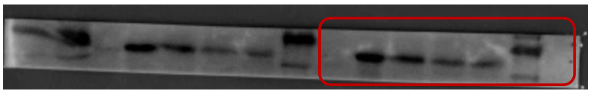</td></tr>
<tr><td colspan="2">Original films of P65 (65KDa) of THP-1 cells.</td></tr>
<tr><td>Bands: Marker, Marker, CT, TNF-α, 200 μM, 150 μM, 100 μM, Marker, CT, TNF-α, 200 μM, 150 μM, 100 μM, Marker</td><td>Bands: Marker, CT, TNF-α, 200 μM, 150 μM, 100 μM, Marker</td></tr>
<tr><td>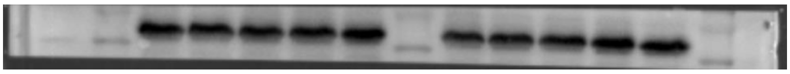</td><td>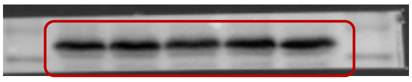</td></tr>
<tr><td colspan="2">Original films of p-GSK3β (46KDa) of THP-1 cells.</td></tr>
<tr><td>Bands: Marker, Marker, CT, TNF-α, 200 μM, 150 μM, 100 μM, Marker, CT, TNF-α, 200 μM, 150 μM, 100 μM, Marker</td><td>Bands: Marker, Marker, CT, TNF-α, 200 μM, 150 μM, 100 μM, Marker, CT, TNF-α, 200 μM, 150 μM, 100 μM, Marker</td></tr>
<tr><td>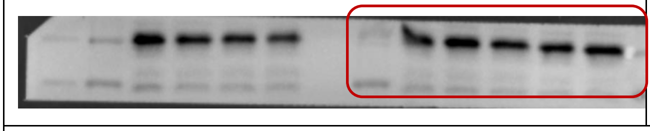</td><td>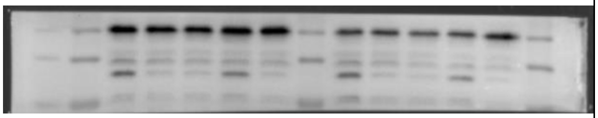</td></tr>
<tr><td colspan="2">Original films of GSK3β (46KDa) of THP-1 cells.</td></tr>
<tr><td>Bands: Marker, Marker, CT, TNF-α, 200 μM, 150 μM, 100 μM, Marker, CT, TNF-α, 200 μM, 150 μM, 100 μM, Marker</td><td>Bands: Marker, Marker, CT, TNF-α, 200 μM, 150 μM, 100 μM, Marker</td></tr>
</table>

<table>
<tr><td>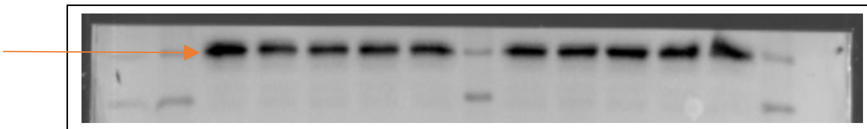</td><td>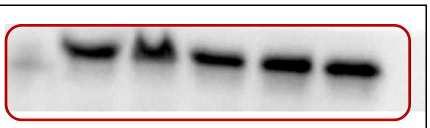</td></tr>
<tr><td colspan="2">Original films of GAPDH (36KDa) of THP-1 cells.</td></tr>
<tr><td>Bands: Marker, Marker, CT, TNF-α, 200 μM, 150 μM, 100 μM, Marker, CT, TNF-α, 200 μM, 150 μM, 100 μM, Marker</td><td>Bands: Marker, Marker, CT, TNF-α, 200 μM, 150 μM, 100 μM, Marker</td></tr>
<tr><td>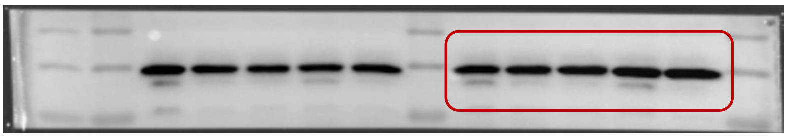</td><td>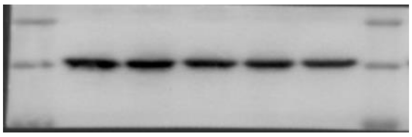</td></tr>
</table>
